# ADHD in Adulthood: Clinical Presentation, Comorbidities, and Treatment Perspectives

**DOI:** 10.3390/ijms262211020

**Published:** 2025-11-14

**Authors:** Ewelina Bogdańska-Chomczyk, Mariusz Krzysztof Majewski, Anna Kozłowska

**Affiliations:** Department of Human Physiology and Pathophysiology, School of Medicine, Collegium Medicum, University of Warmia and Mazury, Warszawska Av, 30, 10-082 Olsztyn, Poland; ewelina.bogdanskachomczyk@student.uwm.edu.pl (E.B.-C.); mariusz.majewski@uwm.edu.pl (M.K.M.)

**Keywords:** adult ADHD, diagnosis, treatment, epidemiology, quality of life, neurobiology, comorbidity, executive dysfunction, pharmacotherapy, cognitive–behavioral therapy

## Abstract

Attention-deficit/hyperactivity disorder (ADHD) in adults has become an increasingly recognized clinical entity, with growing attention in research and healthcare settings. ADHD can significantly affect multiple domains of adult functioning, including education, employment, interpersonal relationships, and both mental and physical health. However, despite the expanding body of literature, gaps in understanding persist. This narrative review synthesizes current evidence on adult ADHD. The literature was systematically searched in databases such as PubMed, Scopus, and PsycINFO using predefined keywords related to ADHD in adults. Inclusion criteria focused on peer-reviewed articles published between 2010 and 2025, addressing epidemiology, etiology, diagnosis, treatment, and functioning. Exclusion criteria included studies with pediatric populations only or lacking methodological rigor. ADHD in adults is prevalent worldwide, with considerable heterogeneity across studies. Its etiology involves complex interactions between genetic, neurobiological, and environmental factors. Clinical presentation in adulthood differs from childhood, with symptoms such as inattention, emotional dysregulation, and executive dysfunction predominating. Diagnostic challenges include retrospective assessment of childhood symptoms and comorbidity with mood and anxiety disorders. Pharmacotherapy and cognitive–behavioral interventions show efficacy, particularly when combined in integrated care models. ADHD negatively affects quality of life and occupational and social functioning and increases the risk of comorbid disorders, including psychoactive substance use. Adult ADHD is a multifaceted condition requiring a comprehensive, multidisciplinary approach to diagnosis and management. Future research should aim to refine diagnostic tools, explore neurobiological markers, and tailor interventions to individual profiles. Expanding knowledge on adult ADHD will improve identification, treatment outcomes, and overall quality of life for affected individuals.

## 1. Introduction

### 1.1. Significance of the Problem

Attention-deficit hyperactivity disorder (ADHD) has been considered a disorder of childhood. However, in recent years, there has been growing scientific and clinical interest in ADHD among adults. This shift reflects a broader recognition that condition symptoms often persist into adulthood and can significantly affect various aspects of life, including education, work, relationships, and overall mental and physical health [1].

Empirical evidence shows that a substantial number of adults meet the criteria for an ADHD diagnosis, suggesting that the condition has often been underdiagnosed or misdiagnosed in this age group. Estimates suggest that adult ADHD affects between 3% and 6% of the population, underscoring the need for better diagnostic awareness and tailored treatment approaches in clinical practice [2,3,4,5].

Most adults diagnosed with ADHD report that their symptoms began in childhood, although these symptoms were often overlooked at the time. Studies indicate that around 60–65% of individuals diagnosed in childhood continue to experience this condition’s symptoms as adults [6,7]. At the same time, many adults are first diagnosed later in life without a formal childhood diagnosis, highlighting significant gaps in early recognition and the need to improve detection of this neurodevelopmental disorder across the lifespan.

Adult ADHD poses notable challenges both clinically and socially. In education, adults with this condition often struggle with focus, organization, and time management, leading to difficulties in learning and completing tasks. These challenges are associated with lower academic performance and higher rates of educational failure compared to neurotypical peers [8,9]. Occupational functioning is also notably affected by adult ADHD. Symptoms such as impulsivity, procrastination, and difficulty meeting deadlines contribute to diminished productivity, increased job instability, and a higher risk of unemployment. Moreover, the complexity of contemporary work environments characterized by multitasking demands and frequent distractions poses additional challenges for adults managing these symptoms [10,11]. On an interpersonal level, this condition is linked to difficulties including impulsivity, impaired sustained attention during conversations, and problems with emotional regulation. These factors contribute to increased conflict and reduced satisfaction in intimate relationships. Individuals with ADHD also show higher rates of relationship dissolution and tendencies toward social isolation, often due to difficulties interpreting social cues [12,13]. Furthermore, this condition is associated with an elevated risk of comorbid psychiatric conditions, such as depression, anxiety, and substance use disorders, all of which complicate treatment and diminish quality of life [8,14]. Emerging evidence also suggests links between the condition in question and metabolic disorders, including obesity, pointing to complex neurobiological interactions between mental and physical health [15]. These findings highlight the importance of adopting integrated, multidimensional therapeutic approaches.

Given the increasing recognition of adult ADHD and the multifaceted nature of the disorder, a systematic review of the literature is warranted. Such an endeavor would integrate existing evidence, delineate critical research gaps, and address inconsistencies across previous studies. By providing a robust evidence base, it would inform diagnostic and therapeutic frameworks, thereby advancing more effective treatment strategies and enhancing the overall quality of care.

### 1.2. Aim of the Study

This review aims to systematically gather and synthesize current scientific literature concerning ADHD in the adult population. The work focuses on key aspects related to the epidemiology, etiology, neurobiology, clinical presentation, diagnostic methods, and available therapeutic options for this disorder. Furthermore, the review aims to identify existing diagnostic and therapeutic challenges, as well as to highlight significant gaps in the current knowledge that warrant further investigation. Emphasis is also placed on the necessity of an integrative approach to the diagnosis and treatment of ADHD in adults, alongside recommendations for future research directions that may contribute to the optimization of clinical care for this patient group.

### 1.3. Methodology

To achieve the objectives of this review, a systematic literature search was conducted focusing on ADHD in the adult population. The search strategy involved querying major medical and psychological databases, including PubMed, Scopus, Web of Science, and PsycINFO, which provide access to a broad range of scientific publications encompassing original research articles, systematic reviews, and meta-analyses. Keywords used for the search included terms related to ADHD and its manifestation in adults, such as “adult ADHD”, “attention-deficit/hyperactivity disorder in adults”, “epidemiology of adult ADHD”, “diagnosis and treatment of adult ADHD”, “neurobiology of ADHD”, “ADHD comorbidity”, and “psychotherapy and pharmacotherapy in adult ADHD”. These keywords were employed both individually and in combination using Boolean operators (AND, OR) to ensure comprehensive retrieval of relevant literature. The review covered publications from the period 2010 to 2025 to incorporate both contemporary findings and seminal studies. This timeframe was selected due to the significant advancements in research on adult ADHD and evolving diagnostic and therapeutic standards.

Inclusion criteria for selected publications were articles published in peer-reviewed scientific journals, studies focusing on adult populations (≥18 years) diagnosed with ADHD, papers addressing epidemiology, etiology, neurobiology, clinical presentation, diagnosis, or treatment of ADHD in adults, and publications written in English or Polish. Exclusion criteria included studies exclusively targeting pediatric or adolescent populations, non-scientific articles such as popular science reports, commentaries, or conference abstracts without full text availability, studies with poor methodological quality or insufficient sample sizes limiting reliable analysis, and publications before the year 2010 that did not contribute significant updated knowledge.

The retrieved articles underwent qualitative synthesis, involving critical analysis of the content with respect to key thematic domains including epidemiology, etiology, neurobiology, diagnosis, treatment, functioning, and quality of life. Where applicable, quantitative data such as prevalence rates, treatment efficacy, and risk factor occurrence were also extracted and considered. Data analysis was conducted narratively, facilitating identification of major trends, scientific consensus, and discrepancies and gaps in the literature. This approach enabled a comprehensive overview of adult ADHD and highlighted areas warranting further investigation to enhance diagnostic accuracy and therapeutic outcomes.

## 2. Epidemiology of Adult ADHD

### 2.1. Prevalence

ADHD is a neurodevelopmental condition traditionally associated primarily with childhood; however, contemporary epidemiological research confirms that ADHD frequently persists into adulthood in a significant proportion of cases. Understanding the prevalence of adult ADHD on both global and regional levels is essential for improving clinical recognition, healthcare planning, and targeted interventions. Epidemiological data indicate that the global prevalence ranges between approximately 2.5% and 6.7%, with an average estimate of around 2.8%, underscoring the disorder’s common occurrence and simultaneous underdiagnosis in adult populations [2,5].

The variability in adult ADHD prevalence is largely influenced by methodological factors and the characteristics of the populations studied. Differences in diagnostic criteria, such as DSM-IV, DSM-5, or ICD-10, as well as the assessment tools employed, ranging from self-report questionnaires to structured clinical interviews, substantially affect reported prevalence rates [16]. Typically, self-reported symptom measures yield higher prevalence estimates due to greater sensitivity but lower specificity compared to clinician-administered diagnostic interviews. Furthermore, the nature of the study population plays a crucial role; clinical samples or individuals seeking psychiatric care generally exhibit higher ADHD rates than community-based samples, reflecting selection bias [17,18]. Age, sex, and socioeconomic factors also contribute to variability, with younger adults and males typically demonstrating higher prevalence. This neurodevelopmental disorder in adult females may be underrecognized due to differing symptom presentations, co-occurring conditions such as anxiety and depression, complicating the diagnostic process and leading to a higher risk of misdiagnosis [19]. Regional and cultural variations, differing mental health awareness, healthcare access, and social stigma further modulate reported prevalence rates [20,21,22]. Additionally, study design elements, including cross-sectional versus longitudinal methodologies, influence the interpretation of ADHD prevalence by either capturing a snapshot or longitudinal persistence of symptoms [23]. Regional variation in the prevalence of ADHD in adults, as shown in Table 1, reflects not so much the biological variability of the disorder as the influence of the systemic, cultural, and methodological factors described above.

A key finding from recent research is that, although diagnostic criteria (DSM/ICD) require the demonstration of symptoms in childhood, the vast majority of adults with ADHD were not diagnosed as children. Retrospective data indicate that more than two-thirds of adults with ADHD report symptoms present in childhood [5,34], highlighting substantial underdiagnosis at early developmental stages. Data from the United States are particularly striking in this regard. The CDC (2023) estimates that 15.5 million adults in the USA (6.0% of the population aged ≥18 years) currently have ADHD; of this group, as many as 55.9% were first diagnosed at age 18 or older [35]. Reviews of clinical records indicate even higher rates: in one study of psychiatric and primary care patients in the USA, only 25% had a childhood diagnosis, suggesting that ~75% received their first diagnosis as adults [36]. A similar pattern, though based on smaller datasets, is observed in other regions. In Japan, an analysis of the national health insurance registry (2010–2019) showed that 40% of all new ADHD diagnoses were made in adults (aged ≥20 years) [37] (see Table 2).

Global statistics and data on late diagnosis also mask significant demographic issues, the most important of which is the systemic underdiagnosis of women. Adult ADHD in women is often unrecognized due to differences in symptom presentation—frequently with a predominance of the inattention component over hyperactivity and greater internalization of symptoms. Moreover, in women, the clinical picture is often complicated by comorbid anxiety and depressive disorders. These symptoms, being more “visible” to clinicians, can obscure underlying ADHD or lead to misdiagnosis (e.g., solely depression or anxiety disorders) [19,38,39]. As a result, while population-based studies typically show higher prevalence in men, this difference may reflect diagnostic bias rather than biology. Staley et al. data support this notion, suggesting that women are diagnosed significantly later: it is estimated that ~61% of women with ADHD were first diagnosed in adulthood, compared with ~40% of men [35].

ADHD rarely occurs in isolation. Up to 50–80% of adults with ADHD meet criteria for at least one other psychiatric disorder [40,41] (Table 3). Notably, recent literature underscores that ASD (Autism Spectrum Disorder) represents one of the most clinically and etiologically significant comorbidities. Across studies, 15–50% of adults with ADHD exhibit autistic traits or meet ASD diagnostic thresholds, while 15–35% of adults with ASD also fulfill ADHD [42,43].

This substantial overlap supports the view that ADHD and ASD exist along a shared neurodevelopmental spectrum, characterized by convergent deficits in executive control, attention regulation, and social cognition [44]. Neuroimaging and polygenic studies demonstrate shared alterations in fronto-striatal and default-mode networks, and overlapping risk loci such as FOXP1 and CNTNAP2 [45,46]. Functionally, adults with co-occurring ADHD–ASD show additive impairments—lower quality of life, greater emotional dysregulation, and poorer occupational and social outcomes than either disorder alone [47].

Clinically, this co-occurrence necessitates integrated diagnostic assessment and multimodal treatment approaches, combining pharmacotherapy for attentional symptoms with neurodiversity-adapted cognitive–behavioral interventions targeting social-communication and emotional-regulation difficulties. The current evidence base supports conceptualizing ADHD–ASD overlap as a distinct hybrid phenotype rather than a mere comorbidity [48,49,50].

The emerging literature supports a paradigm shift from viewing ADHD and ASD as discrete categories to recognizing them as overlapping spectra within a unified neurodevelopmental continuum. The frequent co-occurrence of these disorders, coupled with shared genetic and neurobiological markers, challenges categorical diagnostic frameworks. From a public health standpoint, integrating ADHD–ASD screening into adult psychiatric assessments is essential for early identification and optimized intervention strategies.

**Table 3:** Most common psychiatric comorbidities in adult ADHD.

Disorder Category	Estimated Comorbidity (%)	Clinical Implications (Interpretation)	Source
**Mood Disorders**	30–50%	ADHD symptoms (e.g., emotional dysregulation) may be masked by depression or misdiagnosed as bipolar disorder.	[51,52]
**Anxiety Disorders**	30–50%	Anxiety may be both a consequence (e.g., life failures related to ADHD) and a separate condition. It significantly complicates the clinical picture.	[41]
**Substance Use Disorders (SUD)**	25–50%	Often interpreted as attempts at “self-medication” of ADHD symptoms (impulsivity, attention deficits). Significantly worsens prognosis.	[53,54]
**Personality Disorders**	Increased risk	Shared features (impulsivity, emotional dysregulation), especially with Borderline and Antisocial types, make the diagnostic process extremely complex.	[55]
**Learning Difficulties/Executive Function Deficits**	Frequent	Highlights the neurodevelopmental nature of ADHD; executive function deficits are often at the core of academic and occupational impairments.	[56]
**Autism Spectrum Disorder (ASD)**	15–50%	Reflects shared neurodevelopmental and genetic origins; leads to compounded functional impairment and emotional dysregulation.	[50]

The data presented in Table 3 should not be interpreted as a simple sum of diagnoses, but rather as a dynamic system of mutual interactions. Mood disorders [51,52] and anxiety disorders [41] often exacerbate executive dysfunction associated with ADHD, while the symptoms of ADHD itself (e.g., mood lability, impulsivity) may be misdiagnosed as bipolar disorder or borderline personality disorder [55].

### 2.2. Risk Factors and Predictors

The etiology of ADHD is most accurately conceptualized not as the consequence of a single etiological source but as a multifactorial neurodevelopmental condition arising from the interaction between genetic predisposition and environmental influences (G×E). These factors jointly shape brain development and influence the clinical emergence of ADHD across the lifespan [57,58]. Evidence from twin studies consistently demonstrates a robust genetic contribution to ADHD, with heritability estimates of approximately 74% [57,58]. This places ADHD among the most heritable psychiatric disorders, underscoring inherited neurobiological vulnerability as a central etiological component [50]. Importantly, the genetic influence on ADHD is substantial yet probabilistic—genetic liability increases susceptibility but does not determine the disorder in isolation. Initial molecular studies focused on candidate genes, particularly within dopaminergic and noradrenergic pathways, aligning with known mechanisms of stimulant medications [59]. Variants within the dopamine transporter gene (DAT1/SLC6A3) and the dopamine D4 receptor gene (DRD4, especially the 7-repeat allele) were repeatedly associated with increased ADHD risk [59,60]. These findings formed the basis of the classical dopaminergic hypothesis, emphasizing dysregulation of reward and executive function circuits. Nevertheless, individual genetic variants accounted for only small increments of risk. Subsequent genome-wide association studies (GWAS)—notably those conducted by large consortia such as the Psychiatric Genomics Consortium (PGC)—fundamentally reshaped understanding of ADHD genetics. These studies demonstrate that ADHD is not attributable to a single “ADHD gene,” but rather exhibits a highly polygenic architecture, in which hundreds to thousands of common genetic variants collectively contribute modest but additive effects [59]. Identified loci are enriched in genes expressed in the developing brain and involved in neurodevelopmental processes, reinforcing the biological foundations of the disorder [59]. In sum, genetic vulnerability constitutes the primary predictor of ADHD risk. However, it operates as a cumulative and probabilistic neurodevelopmental liability, which may remain latent or compensated until challenged by environmental or developmental stressors. This perspective explains both individual variability in symptom expression and the heterogeneity observed in clinical presentation.

**Figure 1:**
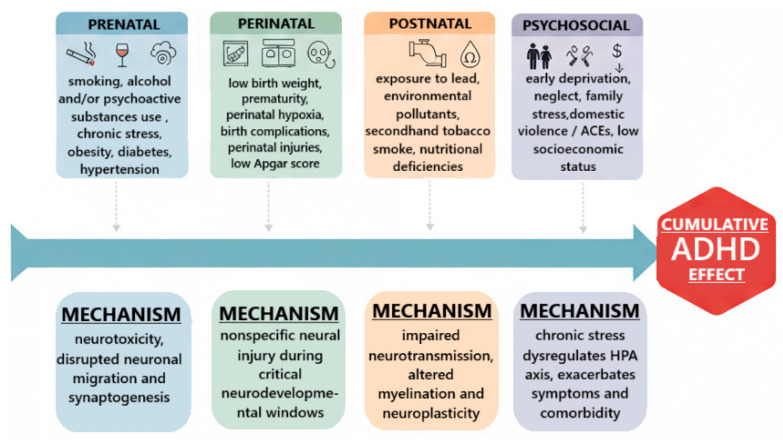
Major environmental risk factors and modulators in ADHD.

While genetics provides a foundation of vulnerability, environmental factors modulate the expression and timing of ADHD onset, particularly among individuals carrying elevated genetic risk. These influences occur across developmental windows. Prenatal and perinatal factors represent key environmental contributors. Maternal smoking [61] and alcohol exposure [62] during pregnancy are associated with increased ADHD risk, likely through neurotoxic effects on fetal development. Prematurity, low birth weight, and perinatal hypoxia have also been linked to subsequent ADHD, suggesting vulnerability due to subtle neural injury during critical periods of maturation [58]. Postnatal exposures include toxic and nutritional factors. Exposure to lead in childhood is associated with increased ADHD symptoms and executive dysfunction [63]. Nutritional insufficiencies, including low levels of omega-3 fatty acids and iron, have been implicated in symptom severity, though findings are less consistent [62]. The psychosocial environment plays a modulating role as well. Early adverse childhood experiences (ACEs), family conflict, low socioeconomic status, and neglect predict more severe ADHD trajectories and greater comorbidity (e.g., anxiety, oppositional symptoms) [64,65]. These exposures are best conceptualized not as primary causes of ADHD but as amplifiers of symptom burden among genetically susceptible individuals. A summary of key environmental risk domains and their putative neurodevelopmental mechanisms is presented in Figure 1.

The convergence of genetic and environmental evidence supports a diathesis-stress model, indicating that neither genetic risk nor environmental exposure alone is sufficient to account for ADHD. Instead, gene-environment interactions (G×E) determine the likelihood, severity, and developmental timing of symptom expression [57]. Environmental influences exert differential effects depending on genotype, such that individuals with specific genetic profiles (e.g., variants in dopaminergic or serotonergic genes such as 5-HTTLPR) are more susceptible to the neurobehavioral impact of early adversity and psychosocial stress [57].

## 3. Etiology and Neurobiology of Adult ADHD

### 3.1. Neurobiological Models

The etiology of adult ADHD is complex, reflecting a significant neurobiological basis shaped by a strong genetic component. It is not a disorder of a single brain region but rather a systems-level dysfunction involving neurotransmitter signaling, structural brain development, and the functional connectivity of large-scale neural networks.

### 3.2. Neurotransmitter System Dysfunctions

The “catecholamine hypothesis” remains the most robust neurochemical model of ADHD, positing a primary dysregulation in dopaminergic (DA) and noradrenergic (NE) signaling. The DA system is central to executive function, motivation, and reward processing. The predominant theory suggests a state of hypodopaminergic functioning, particularly within fronto-striatal pathways. This is supported by molecular imaging (PET/SPECT), which, despite some inconsistencies, meta-analytically points to increased DA transporter (DAT) density in the striatum [66]. Higher DAT availability would lead to excessive reuptake and thus lower synaptic DA levels. A more nuanced interpretation is the “dopamine signaling” hypothesis (also known as the tonic-phasic model). This model proposes that low tonic (baseline) DA levels, particularly in the prefrontal cortex (PFC), impair the optimal “tuning” of cortical networks. This, in turn, results in attenuated phasic (task-related) DA firing in response to salient stimuli, leading to a weakened “signal-to-noise” ratio in the PFC [67]. This weakened signal impairs the maintenance of working memory and attentional focus. Concurrently, dysregulation in the mesolimbic pathway is linked to altered reward processing and the “delay aversion” characteristic of ADHD [68]. The NE system, originating from the locus coeruleus, is critical for modulating arousal, vigilance, and the “top-down” control of executive functions within the PFC. NE modulates the firing of PFC pyramidal neurons via postsynaptic *α2A*-adrenoceptors, enhancing relevant network signals (the “signal”) and suppressing irrelevant ones (the “noise”) [69]. Noradrenergic dysregulation is thought to contribute directly to inattention and emotional lability. The clinical efficacy of NE reuptake inhibitors (e.g., atomoxetine) and *α2A*-agonists (e.g., guanfacine) in treating ADHD underscores the therapeutic importance of this system [69]. Latest evidence suggests a “multi-modular” chemical imbalance. Magnetic Resonance Spectroscopy (MRS) studies have identified alterations in the glutamate-glutamine cycle—the brain’s primary excitatory system—in the PFC and striatum. Given that glutamate signaling is tightly coupled with and modulates DA release, this suggests that fronto-striatal dysfunction in ADHD may be a product of imbalanced glutamate/GABA-DA interactions [70]. The serotonergic (5-HT) system has also been implicated, particularly in relation to impulsivity and the high rates of comorbid mood and anxiety disorders [71].

### 3.3. Structural and Functional Brain Abnormalities

Decades of neuroimaging have provided compelling evidence for subtle but consistent structural differences in the brains of individuals with ADHD. The most robust and replicated findings come from the ENIGMA-ADHD consortium, a massive multi-site meta-analysis. In the largest study to date, individuals with ADHD (both children and adults) showed significantly smaller volumes in key subcortical regions: the basal ganglia (caudate, putamen), nucleus accumbens, amygdala, and hippocampus [72]. These structures are central nodes in fronto-striatal circuits. The findings reinforce the neurobiological basis for reward and motivation deficits, while basal ganglia alterations relate directly to the executive and inhibitory control functions of the fronto-striatal loops. A key neurodevelopmental theory of ADHD is that of a delay in cortical maturation, particularly in the PFC. While this delay (e.g., in reaching peak cortical thickness) is prominent in children [73], findings in adults are more heterogeneous. A follow-up ENIGMA study focusing on cortical metrics found that group differences were less pronounced in adults than in children [72]. This heterogeneity may reflect several factors: compensatory neurodevelopmental mechanisms, the “normalization” of brain structure in those who remit, or the persistence of structural differences only in the most severe adult case. Structural connectivity, as measured by Diffusion Tensor Imaging (DTI), is also compromised. Across the literature, reduced fractional anisotropy (FA)—a hallmark of white-matter disruption—is repeatedly observed in major association and projection tracts [74,75]. These include the corpus callosum (impairing interhemispheric communication), the cingulum bundle (connecting limbic and prefrontal areas), and the superior longitudinal fasciculus, a critical pathway linking frontal and parietal nodes of the fronto-parietal attention networks [74]. This impaired structural connectivity suggests that inefficient information transfer between distal brain regions is a core pathophysiological feature [76]. Often overlooked, the cerebellum is increasingly implicated in the cognitive (not just motor) deficits of ADHD. The cerebellum is reciprocally connected with prefrontal and parietal cortices via fronto-cerebellar loops. It is thought to be crucial for cognitive timing, error processing, and response preparation—all functions impaired in ADHD. Literature data confirm smaller cerebellar volumes, supporting its role in the disorder’s pathophysiology [77].

### 3.4. Aberrant Large-Scale Brain Network Functioning

Neuroimaging research has increasingly highlighted ADHD as a disorder characterized by dysfunctions in large-scale brain networks rather than isolated regional abnormalities.

The Default Mode Network (DMN), which includes the medial PFC, posterior cingulate cortex, and precuneus, is typically active during rest and involved in self-referential thinking. In adults with ADHD, studies have shown hyperconnectivity within the DMN and impaired suppression of its activity during tasks requiring attention, leading to increased mind-wandering and distractibility [78]. This failure to properly deactivate the DMN compromises engagement of attentional networks and is considered a core feature of ADHD symptomatology.

Similarly, the Central Executive Network (CEN), primarily involving the DLPFC and posterior parietal cortex, supports high-order cognitive functions such as working memory and decision-making. Research has demonstrated hypoactivity and reduced connectivity within the CEN in adults with ADHD, which correlates with deficits in executive functioning, cognitive flexibility, and goal-directed behavior [79]. These disruptions contribute to difficulties in organizing and prioritizing tasks, essential for effective everyday functioning.

The Salience Network (SN), composed of the anterior insula and anterior cingulate cortex, plays a critical role in detecting and filtering salient stimuli that require attention. Dysfunctional activity within the SN has been reported in ADHD populations, resulting in inefficient allocation of cognitive resources and exacerbation of inattention and impulsivity symptoms [80]. Impairments in the SN undermine its capacity to facilitate switching between the DMN and task-positive networks, illustrating the complex network-level dysregulation present in ADHD.

The fMRI studies, especially those examining resting-state connectivity, have revealed widespread disruptions in interactions among intrinsic brain networks in ADHD. A meta-analysis identified consistent alterations in connectivity between the DMN and CEN in adults with the disorder, which correlate with attentional deficits and impaired cognitive control [78]. These findings reinforce the view of ADHD as a disorder involving disrupted coordination among multiple brain systems rather than localized brain dysfunction. Moreover, the cerebellum, traditionally associated with motor coordination, has emerged as a significant structure implicated in ADHD-related cognitive deficits. Neuroimaging evidence indicates that individuals with this condition exhibit smaller cerebellar volumes and altered cerebellar connectivity, which likely contribute to attentional lapses and challenges in cognitive regulation [81,82]. This expanding understanding underscores the cerebellum’s role beyond motor function, including its involvement in timing, attention shifting, and executive control, processes commonly impaired in ADHD.

Collectively, these neuroimaging findings emphasize ADHD as a disorder of network dysfunction, involving aberrant activity and connectivity within and between the DMN, CEN, SN, and cerebellar circuits. This network perspective advances the understanding of ADHD pathophysiology and holds promise for informing more precise diagnostic and therapeutic strategies.

### 3.5. Additional Neuroimaging Findings in ADHD

Beyond structural and functional abnormalities identified in cortical and subcortical regions, recent large-scale and multimodal studies have provided additional insights into the neurobiology of ADHD. The ENIGMA-ADHD consortium, using voxel-based morphometry and analyses of cortical thickness, surface area, and volume, revealed reduced surface area in frontal regions, the cingulate gyrus, and temporal areas in children, whereas findings in adults have been more inconsistent, with some studies showing similar reductions and others reporting no significant group differences. Such variability likely reflects the influence of age-related neurodevelopmental trajectories, medication exposure, and the heterogeneity of ADHD across the lifespan [72]. Complementing these structural findings, diffusion tensor imaging (DTI) has highlighted alterations in white matter integrity, with reductions in fractional anisotropy observed across projection, commissural, and association pathways. These disruptions in structural connectivity correlate with symptom severity and cognitive deficits, suggesting that impaired communication between brain regions contributes to attentional and executive dysfunctions [83]. Moreover, electrophysiological studies employing electroencephalography (EEG) consistently report reduced P3 amplitudes during sustained attention tasks in adults with ADHD. The P3 component, an event-related potential reflecting attentional allocation and working memory processes, is diminished in ADHD, indicating difficulties in monitoring and sustaining attention [84]. Taken together, evidence from structural MRI, DTI, and EEG underscores that ADHD is characterized not only by cortical and subcortical abnormalities but also by widespread disruptions in structural connectivity and neurophysiological processing, reinforcing the view of ADHD as a disorder of distributed brain network dysfunction.

In summary, the neurobiology of adult ADHD is best described as a disorder of distributed network integrity. It begins with a high polygenic diathesis, which, when coupled with environmental insults, perturbs the development of critical neurochemical systems, particularly the catecholaminergic modulation of fronto-striatal-cerebellar circuits. This leads to subtle but significant alterations in brain structure (e.g., smaller subcortical volumes, compromised white matter tracts). Ultimately, these structural and chemical abnormalities cascade into the functional domain, manifesting as a dynamic disconnection syndrome. The inefficient “switching” and coordination between the DMN, CEN, and SN result in an unstable cognitive state, impaired executive control, and the core clinical symptoms of the disorder. Emerging research is now probing novel etiological pathways. This includes the role of neuroinflammation, with PET studies showing increased microglial activation in adults with ADHD [85], and the microbiome-gut-brain axis, which may influence both neuroinflammation and neurotransmitter synthesis [86]. The notable neurobiological heterogeneity across individuals also suggests that adult ADHD is likely not a monolithic entity but a heterogeneous clinical syndrome with multiple, potentially overlapping, neurobiological subtypes.

## 4. Neuropsychological Features of Adult ADHD: Comorbidity and Diagnostic Challenges

### 4.1. Neuropsychological Perspective on Symptoms of Adult ADHD

The DSM-5 defines ADHD by two symptom domains: inattention and hyperactivity/impulsivity, each with nine possible symptoms. In adults (age ≥ 17), at least five symptoms in one domain are required for diagnosis (vs. six for children) [87,88]. By these criteria, three presentations are distinguished: predominantly inattentive (ADHD-I), predominantly hyperactive–impulsive (ADHD-HI), and combined (ADHD-C) [87,88]. In clinical and community samples of adults with ADHD, the combined presentation is by far the most common, accounting for roughly 63.5% of cases [89]. The inattentive presentation comprises about 31% of adult cases, and the pure hyperactive–impulsive presentation only about 7% [90]. In practice, this shift reflects the fact that overt hyperactivity often lessens with age, whereas inattentive symptoms (e.g., difficulty initiating tasks, sustaining concentration, forgetfulness, disorganization, lateness) become the predominant features in adult ADHD [89]. Therefore, a comprehensive understanding of adult ADHD requires moving beyond the DSM-5 symptom list to the underlying neuropsychological constructs. The clinical profile is manifesting across two primary, interacting domains: cognitive dysregulation (i.e., executive dysfunction) and emotional dysregulation (see Table 4).

The core attentional and organizational difficulties in ADHD are widely implicated as deficits in executive functions (EFs)—a set of higher-order cognitive processes for goal-directed behavior, including planning, working memory, cognitive flexibility, and inhibitory control [92,93]. Empirical evidence from neuropsychological testing confirms that, as a group, adults with ADHD perform worse than non-clinical controls on tasks assessing these functions. Deficits are frequently observed in sustained and divided attention, planning and set-shifting (e.g., Tower of London), response inhibition (e.g., Continuous Performance Test), and interference control (e.g., Stroop) [92,93,96]. The clinical relevance of these deficits is high; adults exhibiting multiple EF impairments often report greater academic and occupational difficulties [93,97]. However, the magnitude of these executive deficits in adult ADHD is modest and varies widely across individuals [92,98]. On average, adults with the condition perform worse than healthy controls on EF tests, but effect sizes tend to be small to moderate [92]. Many patients perform within normal limits on standard tasks. Crucially, studies have found that lower-level impairments, such as slow processing speed or brief lapses of attention, account for much of the apparent executive dysfunction [98]. For example, deficits in basic processing speed and focused attention explain a large share of the variance in complex EF tasks [98]. In practice, this means that when ADHD adults struggle with, say, planning or working memory tests, it often reflects more elementary attentional constraints and distractibility rather than isolated “executive impairment.” Thus, while planning/organization problems, working memory deficits, inflexibility, and poor impulse control are commonly observed in adult ADHD, the severity of these deficits can differ greatly among patients [92,94].

Adult ADHD is neuropsychologically heterogeneous: not all patients show the same cognitive profile. In group studies, affected adults as a whole score lower on attention and EF tests than healthy controls, but these differences are subtle. One large neuropsychological survey found that effect sizes for differences between diagnosed individuals and controls ranged only from small to medium (Hedges’ g ≈ 0.05–0.70) [92]. Remarkably, about 11% of clinically identified cases performed within the normal range on all cognitive measures [92], and many others had just a few deficit scores. This means that on any given EF test, a large minority will score normally, so cognitive deficits are not universal in the condition.

Moreover, neuropsychological impairments are not specific to this disorder. Studies comparing patient groups—those ultimately diagnosed with ADHD and those evaluated but given alternative diagnoses—show very similar patterns of deficit. Comprehensive clinic-based studies found that outpatients displayed impairments across a broad range of cognitive domains, but so did other psychiatric populations: the profiles of EF weaknesses were almost identical between ADHD and non-ADHD clinical groups. Effect sizes for comparisons were mostly small, and neuropsychological tests showed limited power to distinguish ADHD from other psychiatric referrals [99,100,101,102]. Other research confirms that certain deficits (e.g., slow processing speed or response variability) appear both in this population and in adults with mood or anxiety disorders. In sum, although executive and attentional deficits tend to be more frequent in ADHD than in non-clinical populations, the disorder is not defined by a unique neuropsychological signature. The large individual differences and overlap with other conditions imply that no single cognitive test reliably “diagnoses” it [98].

In addition to cognitive symptoms, many adults experience significant difficulties regulating emotions. Clinicians often observe low frustration tolerance, irritability, mood swings, and temper outbursts, sometimes labeled as emotional dysregulation. For example, patients frequently report becoming quickly angry or upset, then struggling to calm down. Although DSM-5 does not list emotional instability among the core diagnostic criteria, it is a common feature in practice [87]. A recent systematic review estimates that emotional dysregulation is present in roughly 34–70% of adults with the disorder [103]. Moreover, such affective problems tend to be linked with greater overall symptom severity, executive deficits, and comorbid conditions [104]. In other words, individuals who struggle most with emotional control also tend to have more pronounced clinical difficulties and additional psychiatric burdens.

It is now recognized that emotional impulsivity is another important component. Impulsivity is already a formal symptom cluster (e.g., blurting out answers, difficulty waiting for turns), but there is a subtype specifically tied to emotion. Many adults report acting rashly when experiencing intense feelings (sometimes called “negative urgency”), such as making hasty decisions when angry or anxious. These emotion-driven impulses are often intertwined with dysregulated mood, for instance, becoming verbally aggressive during a frustration episode. Despite their impact, these affective symptoms are not included in the standard DSM-5 checklist; instead, the DSM notes them only as associated features. Nevertheless, consensus among clinicians and researchers is that emotion regulation problems and impulsive emotional reactions are extremely common in adults with this disorder [104]. They contribute substantially to functional impairment, affecting relationships, work, and well-being. In summary, adult ADHD typically involves not only inattention and behavioral impulsivity, but also pervasive difficulties in regulating emotions, even though the latter lie outside the formal DSM symptom criteria [105].

Together, these data indicate that adult ADHD reflects a broader dysregulation syndrome encompassing attentional, executive, and emotional control mechanisms. The modest average neuropsychological effects, marked individual variability, and overlap with other psychiatric conditions underscore that no single cognitive test can diagnose ADHD. Clinically, a multimodal evaluation integrating symptom history, functional impairment, developmental course, and contextual factors remains essential.

### 4.2. Comorbidities and Diagnostic Differentiation

Adult ADHD rarely presents as a singular condition. Epidemiological studies consistently show high psychiatric comorbidity, with 50–80% of adults meeting criteria for at least one additional mental disorder [106,107]. These patterns reflect shared genetic vulnerability, overlapping neurobiological mechanisms (including dopaminergic and fronto-striatal dysregulation), and the cumulative functional impact of chronic attentional and emotional dysregulation. Consequently, diagnostic assessments in adulthood must prioritize comorbidity screening and careful longitudinal history-taking to distinguish primary from secondary symptoms.

Mood and anxiety disorders represent the most common comorbidities, affecting ~50% and 20–50% of adults with ADHD, respectively [106]. While these disorders share features such as restlessness, concentration difficulties, and irritability, key distinctions assist differentiation. Depressive and anxiety symptoms are typically episodic, often fluctuating with life stressors, whereas attentional deficits in ADHD are chronic, pervasive, and present across developmental stages [106,107]. Anxiety in ADHD is frequently secondary to sustained executive strain and repeated performance failures, rather than driven by autonomic hyperarousal or anticipatory worry. Co-occurrence is clinically significant; comorbid mood and anxiety disorders are associated with greater functional impairment, slower treatment response, and higher relapse risk. Guidelines recommend treating acute mood or anxiety symptoms first when they impair diagnostic clarity, then reassessing attentional functioning. Persistent executive and organizational deficits after mood stabilization strongly suggest co-existing ADHD [107].

Substance use disorders (SUD) are markedly over-represented, with a twofold increased risk of alcohol or drug dependence and earlier onset of use [14,107]. Impulsivity, emotional dysregulation, and sensation-seeking contribute to initiation and escalation. Comorbidity confers a more severe clinical profile, including higher rates of polysubstance use, emergency visits, suicidality, and treatment dropout [108]. Bidirectional screening is recommended: individuals with ADHD should be assessed for SUD, and those seeking addiction treatment should be screened for ADHD to prevent under-recognition. Emerging evidence indicates that treating ADHD may reduce relapse and improve SUD outcomes, highlighting the importance of integrated care rather than sequential or exclusionary treatment approaches.

Evidence also links ADHD with binge-eating behaviors and bulimia, with a prevalence of 9–30% [109,110]. Impulsivity, emotional dysregulation, and reward-seeking tendencies may contribute to loss-of-control eating. Restrictive disorders, such as anorexia nervosa, are less commonly associated. Clinicians should assess attentional and emotional regulation difficulties in adults presenting with binge eating or chronic dieting failures, and conversely, screen for disordered eating patterns in those with ADHD [109,110].

Personality disorders, particularly borderline and antisocial, frequently co-occur, with estimates suggesting up to 16–38% prevalence [107,111]. Overlapping features—including impulsivity, affective instability, and interpersonal dysregulation—can complicate diagnostic interpretation. Emotional dysregulation intrinsic to ADHD may mimic personality pathology, emphasizing the importance of developmental history and trait persistence when differentiating conditions. Comorbidity predicts more severe impairment and reduced response to medication, and is especially prominent in forensic populations [107].

Comorbidity substantially worsens clinical course, functioning, and safety outcomes, including hospitalization, legal problems, and suicide risk [106]. Notably, many individuals treated exclusively for depression or anxiety continue to experience disabling attentional symptoms, delaying recovery [106,112]. Treatment of ADHD—particularly with stimulants or atomoxetine—may attenuate depressive symptoms and reduce relapse risk in mood and anxiety disorders [113]. As such, early recognition and integrated treatment are essential.

Consensus guidelines (e.g., NICE NG87; European ADHD Guidelines) emphasize comprehensive diagnostic evaluation, including routine screening for depression, anxiety, SUD, and personality pathology [58,114]. Acute psychiatric conditions (e.g., psychosis, mania, active substance withdrawal) should be stabilized before initiating ADHD pharmacotherapy. In bipolar disorder, mood stabilization precedes cautious use of stimulant or non-stimulant medication [106]. Combined pharmacological and psychosocial treatment, including cognitive–behavioral therapy (CBT) and skills training, yields the most robust outcomes.

### 4.3. Diagnostic Approaches and Challenges

DSM-5 conceptualizes adult ADHD as a persistent neurodevelopmental disorder characterized by inattention and/or hyperactivity–impulsivity leading to significant functional impairment. For individuals aged ≥17 years, endorsement of at least five symptoms in one or both domains for ≥6 months is required [115], reflecting the developmental attenuation of overt hyperactivity relative to childhood [115,116]. The criteria also mandate symptom emergence before age 12 and cross-situational pervasiveness, underscoring ADHD’s early-onset and context-general nature [115,116]. DSM-5 further delineates three clinical presentations—predominantly inattentive, predominantly hyperactive–impulsive, and combined type—which, while useful descriptively, may show fluidity across development and do not always map cleanly onto functional profiles in adulthood. Thus, the diagnostic framework provides essential structure but is limited in capturing heterogeneity in adult symptom trajectories and compensatory behaviors.

In clinical practice, diagnosing ADHD in adults is challenging because symptoms often manifest less as overt hyperactivity and more as internal restlessness, emotional dysregulation, and executive dysfunction—features that may be misinterpreted as primary anxiety or mood disorders. Many adults initially present for treatment of comorbid conditions, particularly depression, anxiety, or substance use disorders [117]. This presentation pattern contributes to frequent diagnostic delay and highlights the need to evaluate ADHD not only by symptom count but by the pattern, context, and functional consequences of symptoms over time. Accurate diagnosis, therefore, requires more than checklist application; clinicians must evaluate symptom persistence, functional impairment, developmental history, and differential diagnoses, consistent with guidance such as NICE NG87, which emphasizes specialist assessment and cross-domain evaluation [116].

Screening instruments play a useful but adjunctive role. The Adult ADHD Self-Report Scale (ASRS) and its short forms provide efficient identification of probable cases and demonstrate strong specificity and reliability (~0.90 Cronbach’s alpha) [31,118]. However, their moderate sensitivity (~69%) means that negative screening results should not exclude diagnosis in clinically suspected cases. Likewise, retrospective instruments such as the WURS-25 offer strong discriminative validity, particularly versus mood disorders, but rely on self-report and are susceptible to recall bias. Structured diagnostic interviews, particularly the DIVA-5, enhance diagnostic precision by systematically assessing developmental history and functional impairment across life domains [119,120]. Together, these tools illustrate that adult ADHD assessment benefits from an integrated psychometric approach, where screening identifies risk, but structured evaluation and clinical judgment confirm diagnosis.

Verification of childhood onset remains a defining but often difficult component of adult ADHD assessment. While collateral information, such as parent reports and school records, enhances validity [117,121], such data are frequently unavailable in routine care. In these cases, diagnostic accuracy relies on triangulation of available evidence, critical appraisal of retrospective self-report, and careful exclusion of alternative explanations such as chronic stress, sleep disorders, or substance use [117,121]. Taken together, optimal diagnostic practice involves a multi-method, multi-informant strategy integrating self-report, clinician-rated interviews, collateral information, and functional evaluation. This approach mitigates risks of over- or under-diagnosis associated with reliance on a single information source and aligns with the developmental and dimensional nature of ADHD [117,121].

In summary, DSM-5 provides a necessary structural anchor for diagnosing adult ADHD, but clinical interpretation must extend beyond symptom counts. A nuanced diagnostic process—attentive to developmental history, compensatory mechanisms, comorbidities, and functional impairment—is essential to differentiate ADHD from overlapping psychiatric conditions and to ensure valid identification of a disorder that remains frequently under-recognized in adulthood.

## 5. Sex-Dependent Differences in Adult ADHD

### 5.1. Epidemiology and Diagnosis

Across epidemiological studies, women are consistently diagnosed with ADHD later in life than men, despite reporting comparable ages of symptom onset [122]. This diagnostic lag appears less related to true differences in developmental trajectory and more to systematic under-recognition of female presentations. Whereas disruptive behaviors in boys typically prompt early referral, girls’ symptoms often manifest as inattention, internalizing distress, and compensatory over-control, which attracts less clinical attention and may be misattributed to mood or anxiety disorders. Notably, DSM-5 presentation categories do not differ meaningfully by sex in adulthood [122], suggesting that diagnostic inequity is not driven by subtype distribution, but by how symptoms are expressed, perceived, and interpreted in clinical and social contexts.

Women also report greater global symptom severity and markedly higher rates of internalizing comorbidities, particularly depression and anxiety [122]. In contrast, men demonstrate higher rates of substance use and externalizing behaviors [123,124]. These patterns reinforce a gendered diagnostic pathway: externalizing symptoms increase detection in men, whereas internalizing profiles in women delay recognition. As a result, sex differences in prevalence reported in clinical samples may partly reflect ascertainment bias, underscoring the importance of gender-sensitive assessment practices.

### 5.2. Clinical and Psychosocial Differences

Sex-dependent symptom profiles extend beyond diagnostic timing. Clinical studies show that adult women with ADHD frequently report greater emotional lability, irritability, chronic overwhelm, and relational strain [125]. The subjective burden tends to be substantial, and internal distress is often accompanied by perfectionistic compensation and high self-criticism—strategies that may temporarily preserve functioning but obscure underlying impairment and delay diagnosis.

Conversely, men more often present with impulsivity, risk-taking, and disruptive externalizing behaviors [126]. These divergent trajectories shape clinical engagement: women frequently enter care through mood or anxiety pathways, whereas men are more often referred due to behavioral or substance-related difficulties [125,126]. Together, these findings indicate that sex differences in adult ADHD should be understood not as categorical distinctions but as probabilistic patterns shaped by neurobiological liability, gendered socialization, and healthcare system biases.

### 5.3. Neurobiological Correlates

Neuroimaging research supports subtle but meaningful sex-related neurobiological variation in ADHD. While adults with ADHD show reduced fronto-striatal and cerebellar gray matter volume overall [127,128,129,130], these reductions appear more robust in males [131,132,133]. Women demonstrate more variable patterns, with relatively preserved fronto-striatal volume but more frequent alterations in limbic and parietal circuitry linked to emotional and attentional regulation [134]. Functional imaging similarly points to sex-differentiated profiles: men typically exhibit reduced activation in executive networks, whereas women often display activation closer to neurotypical controls—potentially reflecting compensatory neural recruitment [135].

Resting-state studies reveal parallel patterns in network connectivity, with women showing more alterations in emotion-related circuits and men in motor–executive pathways [134]. Neurochemical and genetic studies further suggest heterogeneity: men may carry a greater burden of rare structural variants, whereas women may require a higher polygenic threshold to manifest ADHD [136]—supporting a proposed female protective effect. Moreover, fluctuations in ovarian hormones influence dopaminergic and noradrenergic signaling, with clinical reports describing symptom worsening during low-estrogen phases and across reproductive transitions [137,138,139,140]. Collectively, these findings point to sex-specific neurobiological vulnerability and compensation profiles, which may help explain distinct clinical phenotypes.

### 5.4. Diagnostic and Therapeutic Implications

These sex-dependent features have clear diagnostic and therapeutic consequences. Delayed identification in women is associated with prolonged functional impairment, lower self-esteem, and greater psychiatric comorbidity [125]. Improving detection requires active inquiry into subtle inattention, internal distress, and compensatory behaviors, rather than reliance on disruptive symptoms typical of male presentations.

Treatment should similarly be tailored. Emotional dysregulation and internalizing burden often require integrated management of ADHD and mood/anxiety symptoms [125], and addressing ADHD symptoms can itself mitigate secondary depression [141]. Hormonal influences warrant attention: symptom exacerbation premenstrually, postpartum, and during the menopause may require adjustments to stimulant timing or adjunctive strategies [137]. While more empirical data are needed, these observations highlight the need for individualized, phase-informed treatment planning, particularly for women. Ultimately, integrating sex-specific insights into assessment and care can reduce misdiagnosis, optimize therapeutic response, and better reflect the heterogeneity of adult ADHD across the lifespan [137].

## 6. Treatment of Adult ADHD

Adult ADHD is increasingly conceptualized as a chronic neurodevelopmental disorder requiring comprehensive and sustained therapeutic intervention. Unlike pediatric cases, adult presentations frequently involve persistent impairments in executive functioning, including organization, sustained attention, impulse control, and emotional regulation, all of which can adversely affect occupational, social, and emotional domains. When untreated, ADHD in adults is associated with significant psychological and societal burden and a heightened risk of psychiatric comorbidities such as mood disorders, anxiety, and substance use [58].

Management strategies are typically individualized and encompass both pharmacological and psychotherapeutic components. Robust evidence supports the efficacy of stimulant medications (e.g., methylphenidate, amphetamines) and non-stimulants (e.g., atomoxetine) in reducing core symptoms of ADHD [142,143]. Concurrently, CBT (cognitive–behavioral therapy) has emerged as a valuable addition to adjuvant ADHD therapy, facilitating the development of compensatory strategies, improved self-management, and enhanced emotional regulation [144].

### 6.1. Pharmacotherapy

#### 6.1.1. Stimulant Medications

Stimulant medications remain the first-line and most efficacious pharmacological option for adult ADHD, with extensive evidence supporting their ability to reduce core symptoms of inattention, hyperactivity, and impulsivity. These agents fall into two primary categories: amphetamine-based compounds (e.g., dextroamphetamine, lisdexamfetamine) and methylphenidate formulations, both acting primarily through enhancement of catecholaminergic neurotransmission in prefrontal and striatal regions critical for attention and executive control. By inhibiting DA and NE reuptake and facilitating presynaptic release, stimulants normalize the deficient cortical arousal and signal-to-noise ratios characteristic of ADHD [145].

Meta-analytic data confirm robust efficacy in adults, with effect sizes comparable to pediatric populations [146]. Lisdexamfetamine, a prodrug formulation, offers particularly stable plasma concentrations and extended therapeutic coverage, supporting symptom control across occupational and social contexts [147]. Detailed comparisons of efficacy are provided in Table 5. Importantly, treatment response tends to vary between methylphenidate and amphetamine classes, with some adults responding preferentially to one mechanism, underscoring the need for empiric, sequential titration in clinical practice. Although generally well tolerated, stimulants are associated with predictable side effects, most notably insomnia, appetite suppression, anxiety, and mild increases in heart rate and blood pressure [148]. These effects are usually dose-dependent and manageable, but rare cardiovascular events necessitate risk stratification and ongoing monitoring, especially in patients with preexisting cardiac conditions [149]. Comparative safety and pharmacological profiles are summarized in Table 6. Concerns regarding misuse and diversion remain relevant, particularly among young adults and university populations [150]. Nonetheless, the risk is largely contextual: when prescribed within structured monitoring frameworks, stimulants demonstrate low rates of misuse in clinical cohorts. Best practice emphasizes comprehensive diagnostic assessment, education on misuse risks, and judicious prescribing with periodic review [151]. Current guidelines, including NICE NG87, advocate a multimodal approach in which stimulant pharmacotherapy is complemented by psychoeducation, cognitive–behavioral interventions, and skills-based training [116,152]. This combination not only enhances adherence and coping but also targets executive dysfunction and emotional dysregulation—domains less directly addressed by medication.

Increasing emphasis on personalized medicine reflects recognition that adult ADHD is heterogeneous. Optimal pharmacotherapy should consider individual symptom profiles, comorbidities (e.g., anxiety, substance use, sleep disorders), lifestyle, and treatment history [153]. Shared decision-making between clinician and patient strengthens engagement, reduces stigma, and promotes sustained benefit.

#### 6.1.2. Non-Stimulant Medications

Nonstimulant agents—atomoxetine, guanfacine, and viloxazine—constitute vital alternatives for adults who do not respond to, cannot tolerate, or are at risk for misuse of stimulants. Unlike stimulants, these medications act through indirect modulation of catecholaminergic signaling, providing options for patients with differing neurobiological or clinical profiles.

Atomoxetine, a selective norepinephrine reuptake inhibitor (NRI), enhances NE transmission in prefrontal circuits regulating attention and inhibition [154,155]. Its gradual onset of action and absence of dopaminergic stimulation make it particularly suitable for individuals with a history of substance misuse.

Guanfacine, an α2A-adrenergic receptor agonist, improves prefrontal cortical connectivity by strengthening noradrenergic tone and postsynaptic receptor signaling, thereby enhancing working memory and impulse control [154,155]. Its sedative and hypotensive effects may provide added benefit for patients with comorbid anxiety or insomnia.

Viloxazine, a newer nonstimulant, acts as a norepinephrine transporter inhibitor with secondary serotonergic modulation, suggesting potential advantages in addressing emotional dysregulation [156]. Although adult data are still emerging, early findings indicate promising tolerability and symptom reduction.

The efficacy of these agents is supported by multiple randomized controlled trials and meta-analyses. Atomoxetine consistently yields moderate symptom reduction across age groups [157], while guanfacine improves attention and behavioral regulation in adults with emotional dysregulation [154,155]. Preliminary studies of viloxazine report similar benefits, though confirmatory research in adult populations remains needed [157].

Safety and tolerability profiles differ across nonstimulants but are generally favorable. Atomoxetine’s most frequent adverse effects include fatigue, appetite loss, and gastrointestinal discomfort; guanfacine may cause hypotension or rebound hypertension if discontinued abruptly [155]; and viloxazine may induce mild somnolence [156]. Importantly, none are associated with abuse potential—an advantage that facilitates use in populations at risk for substance misuse [154,157].

From a therapeutic standpoint, nonstimulant medications play a strategic role in individualized management, either as monotherapy in patients with stimulant contraindications or as adjuncts to enhance emotional regulation. Tailoring pharmacotherapy to comorbidity patterns—such as anxiety, insomnia, or cardiovascular disease—enhances both safety and efficacy [155,158].

### 6.2. Psychological and Behavioral Interventions

While pharmacotherapy remains foundational, psychosocial interventions are crucial in addressing the functional impairments—organizational deficits, emotional dysregulation, interpersonal conflict, and occupational dysfunction—that persist even with optimal medication. Psychological approaches provide compensatory frameworks that strengthen self-regulation, executive control, and coping.

CBT is the most extensively validated modality for adult ADHD, demonstrating moderate-to-large effects on symptom reduction and comorbid anxiety and depression [159]. By targeting maladaptive cognitions and behaviors, CBT enhances self-management, goal-setting, and time organization [160]. Mechanistically, CBT is thought to promote prefrontal top-down regulation and reduce avoidance behaviors that perpetuate inattention and procrastination.

Dialectical Behavior Therapy (DBT) extends this approach to the domain of emotional dysregulation, emphasizing mindfulness, distress tolerance, and interpersonal effectiveness [161]. Although initially developed for borderline personality disorder, DBT principles are increasingly applied to ADHD, where emotional impulsivity contributes significantly to impairment.

Schema Therapy (ST) addresses deeply rooted maladaptive schemas related to chronic failure, inadequacy, and rejection, which are prevalent in adults with ADHD [162,163]. By restructuring these cognitive–emotional patterns, ST can reduce chronic shame and improve relational functioning—areas often resistant to conventional CBT.

Social Skills Training (SST) specifically targets interpersonal communication and behavioral self-regulation through modeling and feedback [164]. Given the high rates of social conflict and miscommunication in adults with ADHD, SST facilitates the acquisition of adaptive interactional patterns and reduces social anxiety.

ADHD coaching offers a pragmatic complement to psychotherapy, focusing on the real-world application of executive skills. Coaching interventions emphasize structured goal-setting, accountability, and reinforcement of adaptive routines [165]. Evidence indicates significant gains in functional outcomes, self-efficacy, and daily structure when coaching is integrated into comprehensive treatment plans.

Collectively, these interventions highlight that behavioral and cognitive therapies address distinct but interlocking components of ADHD—executive dysfunction, emotional dysregulation, and maladaptive self-concept—thereby extending the benefits of pharmacotherapy and promoting durable functional recovery.

### 6.3. Supportive Treatments

Supportive interventions further enhance outcomes by promoting self-understanding, social connectedness, and self-regulation. Psychoeducation forms the cornerstone of these approaches, providing patients (and often their families) with knowledge about ADHD’s neurobiological underpinnings, symptom expression, and evidence-based treatments. This understanding fosters self-advocacy, treatment adherence, and reduced stigma [166].

Support groups create structured environments for peer exchange and normalization of experience, reducing isolation and enhancing resilience [167]. Participation in such groups is associated with improved mood, self-esteem, and coping capacity.

Mindfulness-based interventions, including Mindfulness-Based Cognitive Therapy (MBCT), cultivate present-focused attention and nonjudgmental awareness. By enhancing metacognitive monitoring, mindfulness practices strengthen executive control and reduce emotional reactivity. Clinical studies demonstrate meaningful reductions in ADHD symptom severity, impulsivity, and comorbid anxiety, alongside improvements in quality of life [168].

Neurofeedback represents a biologically grounded intervention leveraging operant conditioning to enhance regulation of neural oscillations associated with attention and cognitive control. Meta-analyses report moderate efficacy, particularly for attentional domains, though methodological heterogeneity limits definitive conclusions [169]. The approach may complement pharmacotherapy by promoting enduring neuroplastic adaptation.

Finally, ADHD coaching, when used in supportive contexts, provides individualized, goal-oriented behavioral scaffolding that helps translate therapeutic insight into daily practice. Regular feedback loops between coach and client reinforce accountability, enhance executive functioning, and sustain behavioral change [170].

In summary, the growing evidence base underscores that effective adult ADHD management is multidimensional, integrating pharmacological, psychological, and supportive strategies. Stimulants and nonstimulants address neurochemical dysregulation, while psychosocial and behavioral therapies remediate cognitive, emotional, and interpersonal deficits. When combined and personalized, these modalities yield the most durable improvements in symptom control, psychosocial functioning, and overall quality of life. Future research should prioritize longitudinal outcomes, cost-effectiveness, and implementation strategies to broaden accessibility to comprehensive, individualized ADHD care.

**Table 5:** Comparative efficacy of pharmacological and psychological interventions for adult ADHD: pooled effect estimates from meta-analyses and network meta-analyses, expressed as standardized mean difference (SMD) or mean difference (MD) with 95% confidence intervals (CI), based on randomized controlled trials (RCTs). Sample sizes (N) and certainty of evidence graded according to the Grading of Recommendations, Assessment, Development and Evaluations (GRADE) system are reported. CBT is included among psychological interventions.

Intervention	Outcome Measure	Summary Effect (SMD/g)	95% CI	N (Adults)	Quality (GRADE)	Clinical Interpretation
**Amphetamines** (stimulant, e.g., lisdexamfetamine, dextroamphetamine)	Clinician-rated ADHD symptoms (≈12 weeks) [142]	SMD − 0.79	−0.99 to −0.58	*n* ≈ 8131	Moderate–High	Large effect on core ADHD symptoms; rapid onset; guideline first-line [142]
**Methylphenidate** (stimulant)	Clinician-rated ADHD symptoms (≈12 weeks)	SMD − 0.49	−0.64 to −0.35	*n* ≈ 2600	Moderate–High	Moderate effect size; widely recommended as first-line [142]
**Atomoxetine** (non-stimulant)	Clinician-rated ADHD symptoms (10–12 weeks; longer-term effects accrue)	SMD − 0.45	−0.58 to −0.32	*n* ≈ 2050	Moderate	Small–moderate effect; slower onset; useful when stimulants contraindicated or misuse risk [142,171]
**Cognitive–Behavioral Therapy** (adjunct or monotherapy)	ADHD symptoms and functional outcomes	Hedges’ g ≈ 0.65 vs. control	0.44 to 0.86	*n* ≈ 896	Moderate	Moderate benefit for symptoms/function; smaller vs. active controls; valuable for residual deficits [172,173]

**Table 6:** Comparative safety and tolerability profiles of pharmacological treatments for ADHD in adults; incidence of common adverse events, cardiovascular and hepatic signals, abuse potential, and recommended clinical monitoring (Based on Food and Drug Administration Access Data [174,175,176,177]).

Adverse Effect/Safety Domain	Lisdexamfetamine	Methylphenidate	Atomoxetine	Viloxazine	Clinical Action/Monitoring
**Insomnia/sleep disturbance**	**27%** (N = 358; insomnia 27% vs. placebo 8%) [174].	**12.3%** (CONCERTA adults; insomnia 12.3% vs. placebo 6.1%) [178].	**15%** (STRATTERA adults; insomnia 15% vs. placebo 8%) [176].	**23%** insomnia (Qelbree adults; 23% vs. placebo 7%).	Ask about dosing time; prefer morning dosing for stimulants; counsel on sleep hygiene; consider switching to less activating agent or non-stimulant if persistent. Monitor sleep and daytime function.
**Decreased appetite/anorexia/weight decrease**	**Decreased appetite 27%**; decreased weight 3% (adult ADHD Study 7) [174].	**Decreased appetite 25.3%**; weight decreased 6.5% (CONCERTA adults) [175].	**Decreased appetite 16%**; weight decreased 2% (STRATTERA adults) [176].	**Decreased appetite 10%** (Qelbree adults).	Baseline weight; periodic weight monitoring (especially early treatment/children); dietary counselling; assess for clinically significant weight loss → consider dose change/alternative.
**Dry mouth/xerostomia**	**26%** (VYVANSE adults) [174].	**14.0%** (CONCERTA adults) [175].	**20%** (STRATTERA adults) [176].	**10%** (Qelbree adults) [177]	Encourage fluids, sugar-free gum/lozenges, oral hygiene; consider symptomatic measures.
**Nausea/GI adverse events**	**Nausea 7%** (adult ADHD Study 7) [174].	**Nausea 12.8%** (CONCERTA adults) [175].	**Nausea 26%** (STRATTERA adults) [176].	**Nausea 12%** (Qelbree adults) [177].	Take with food if tolerated; antiemetic strategies rarely needed; monitor for persistent GI symptoms and weight loss; check labs if indicated.
**Headache**	Headache reported (adult ADHD trial: ~1% leading to discontinuation; post-market data show higher rates in other indications)—see label [174].	**Headache 22.2%** (CONCERTA adults) [175].	**Headache 19%** (STRATTERA adults table) [176].	**Headache 17%** (Qelbree adults) [177].	Symptomatic treatment (analgesia); monitor for severe or new/worsening headaches—consider cardiovascular assessment if concerning features.
**Cardiovascular (↑ HR, ↑ BP, tachycardia, palpitations)**	**Increased HR 2%**; **↑ BP 3%**; palpitations 2% in adult ADHD Study; note higher heart-rate signals in some adult BED/adult flexible-dose studies [174].	**Tachycardia 4.8%**; palpitations 3.1% (CONCERTA adults); mean small increases in BP/HR reported [175].	**Palpitations 3%**; some adults experienced potentially clinically important HR/BP changes (~5–10% in some analyses); label recommends monitoring [176].	**Tachycardia 4%** (Qelbree adults; also 29% had >20 bpm increases at any time point in adult flexible-dose trial—see label) [177].	Baseline CV history, baseline BP and HR prior to initiation, recheck after titration and periodically; avoid stimulants in uncontrolled CVD; cardiology referral if symptomatic or marked changes. Consider non-stimulant in high CV risk.
**Somnolence/sedation/fatigue**	Somnolence reported infrequently in adult ADHD Study 7 (labels show low % in that adult ADHD study), but somnolence common in some adult BED trials [174].	Sedation not a leading AE for methylphenidate ER in adults (some sedation/sleep disruption reported) [175].	**Somnolence 8%** (STRATTERA adults) [176].	**Somnolence 6%** (Qelbree adults) [177].	Counsel re: driving/operating machinery especially with guanfacine and viloxazine; consider dosing time adjustments; avoid co-administration with other sedating meds where possible.
**Abuse/diversion potential**	**High (controlled substance, Schedule II)**—lisdexamfetamine associated with misuse/diversion risk; note prodrug reduces immediate abuse potential but controlled substance status remains [174].	**High (methylphenidate-controlled substance)** risk of misuse/diversion; label warns about dependence [175].	**Low (atomoxetine not a controlled substance)**; low abuse potential [176].	**Low** (Qelbree/viloxazine not scheduled; low abuse potential) [177].	Screen for current or past SUD at baseline; if active SUD, prefer non-stimulants (atomoxetine, viloxazine) and structured SUD care; if stimulants required, use ER formulations, close monitoring and controlled dispensing.
**Hepatic injury/LFT signals**	Rare post-marketing hepatotoxicity signals are possible; follow label for post-marketing reports [174].	Rare; monitor if clinically indicated [175].	**Labeled: rare cases of severe hepatic injury reported**—monitor (STRATTERA label includes hepatic injury warnings) [176].	Limited long-term adult data; monitor per label; follow LFTs if clinically indicated [177].	Obtain baseline LFTs if liver disease or symptoms; for atomoxetine consider LFT monitoring if symptoms or risk factors (per label).

### 6.4. Dietary Interventions in ADHD Therapy

ADHD is a condition that often persists into adulthood and can significantly affect occupational, social, and emotional functioning. While pharmacotherapy remains the first-line treatment, dietary interventions are increasingly explored as supportive strategies for symptom management.

Among the most studied interventions is supplementation with omega-3 fatty acids, particularly eicosapentaenoic acid and docosahexaenoic acid. These nutrients support neuronal membrane fluidity and synaptic plasticity, both crucial for cognitive control and attention regulation [179]. A systematic review by Catalá-López et al. [180] supports their efficacy in children, though evidence in adults remains less conclusive. Crescenzo et al. [181] also note modest improvements in adults with ADHD but emphasize the need for more rigorous trials.

Micronutrient deficiencies have also been linked to ADHD symptoms. Zinc, a cofactor in neurotransmitter metabolism, is frequently reported at lower levels in individuals with the condition, although the direct impact of supplementation in adults is still under investigation [182]. Magnesium has shown promise in reducing hyperactivity and inattention in children, but adult data are limited [182]. Iron, particularly low ferritin levels, has been associated with reduced dopaminergic activity; supplementation may improve concentration and executive functioning in those with iron deficiency [183]. In turn, B vitamins—including B6, B12, and folate—play critical roles in neurotransmitter synthesis, but their specific effects on ADHD in adults remain unclear [184].

Restrictive elimination diets, typically involving the removal of artificial food additives, preservatives, or common allergens (e.g., gluten or dairy), have been more extensively studied in pediatric populations. However, emerging research suggests they may benefit adults as well. These diets aim to reduce systemic inflammation and identify food-based symptom triggers [185]. Pelsser et al. [182] report that adults who adhered to elimination diets experienced improvements in attention and impulse control, though these findings require confirmation in larger, well-controlled studies.

Overall dietary patterns are another area of growing interest. The Mediterranean diet—rich in vegetables, fruits, whole grains, fish, and olive oil—has been associated with improved cognitive and emotional outcomes. Its anti-inflammatory properties may explain observed reductions in ADHD symptoms [186]. Similarly, the DASH (Dietary Approaches to Stop Hypertension) diet, which emphasizes whole foods and low sodium, has shown potential cognitive benefits in preliminary studies [187].

Implementing dietary interventions effectively requires individual assessment and personalization. Tailoring strategies based on the patient’s dietary habits, nutritional status, and clinical profile is essential for optimizing outcomes [188]. Multidisciplinary collaboration, involving healthcare providers such as dietitians and mental health professionals, enhances intervention design and adherence [189]. Ongoing monitoring of symptoms and nutritional parameters allows for the adaptation of dietary strategies over time, improving both effectiveness and patient engagement [190].

## 7. Psychosocial Functioning and Quality of Life in Adults with ADHD

### 7.1. Occupational and Educational Performance

Adults with ADHD consistently exhibit marked impairments in educational and occupational functioning, with consequences that extend across the lifespan. Numerous reviews and large-scale cohort studies demonstrate persistent educational underachievement, including lower rates of high school and college completion and reduced attainment of advanced degrees compared with controls [191,192]. These academic shortfalls often translate into long-term vocational disadvantages: adults with ADHD more frequently occupy lower-level positions, experience greater employment instability, and earn less income [191,193]. Importantly, these effects are not merely secondary to comorbid conditions but appear to reflect core cognitive and behavioral deficits associated with ADHD itself.

At the workplace level, performance deficits are well documented. Employees with ADHD consistently receive lower productivity ratings, exhibit higher absenteeism, and demonstrate shorter job tenure [10,194]. In a large cross-national survey, workers with ADHD averaged 8.4 excess sick days per year and displayed significant reductions in both work quantity and quality [194]. Epidemiological cohorts corroborate these findings, revealing higher rates of job quitting and dismissal [195] and a pattern of recurrent “job shifting”. For example, a Swedish registry study reported a small but statistically significant increase in annual job changes (incidence rate ratio ≈ 1.03) among adults with ADHD relative to matched controls [195]. Collectively, these data underscore that ADHD in adulthood is not simply a residual childhood condition but a major determinant of lower educational attainment, unstable employment, and diminished productivity [192,195].

Beyond descriptive findings, research increasingly points to specific mechanistic pathways underlying these impairments. Executive dysfunction, particularly deficits in working memory, planning, and cognitive flexibility, emerges as a central explanatory factor [10,196,197]. Poor working memory undermines the ability to retain multi-step instructions, while difficulties in planning and organization compromise task sequencing and prioritization. Consequently, individuals may struggle to meet deadlines or adjust to changing demands, which manifests as missed appointments, incomplete projects, and disorganized schedules [10]. These patterns are empirically supported: workplace studies show that impairments in time management and self-organization mediate the link between ADHD symptoms and job burnout, suggesting that executive dysfunction operates as a proximal driver of occupational underperformance [10,97,198].

Affective and interpersonal difficulties also contribute to functional impairment. Emotional impulsivity and low frustration tolerance can precipitate interpersonal conflict, while inattention may be misinterpreted as disinterest or irresponsibility [10,97,198]. Consequently, adults with ADHD often report strained relations with supervisors and colleagues, further diminishing job satisfaction and career progression.

Despite these challenges, a subset of adults with ADHD demonstrates notable resilience and adaptive functioning. Qualitative studies highlight the use of compensatory strategies such as external structuring systems (e.g., digital calendars, multiple alarms, and task-specific reminders) and behavioral techniques like stimulus control or task segmentation [199]. These self-devised approaches reflect a form of “cognitive offloading,” wherein individuals rely on environmental scaffolds to compensate for executive deficits. Increasingly, formalized interventions such as ADHD coaching have been developed to systematize these strategies. Evidence suggests that structured coaching programs can significantly improve goal attainment, organization, and functional outcomes [200]. Importantly, such approaches embody a shift from deficit-focused to capability-enhancing models, aligning with current trends toward personalized and strengths-based ADHD management.

In summary, ADHD exerts a pervasive and multifaceted impact on educational and occupational functioning, mediated primarily by executive and emotional dysregulation. While pharmacological and behavioral interventions can attenuate symptoms, effective long-term management in adulthood requires targeted support for planning, organization, and self-regulation skills, ideally within occupational and educational settings. Future research should further clarify how individual compensatory mechanisms and environmental accommodations can optimize vocational outcomes for adults with ADHD.

### 7.2. Social Relationships and Family Life

Interpersonal functioning in adults with ADHD is often profoundly affected, with difficulties spanning romantic partnerships, family dynamics, and social networks [195]. Core symptoms such as impulsivity, inattention, and emotional dysregulation manifest directly in relational contexts: impulsivity can lead to hasty decisions or conflictual exchanges, while inattention may be perceived as disengagement, fueling frustration in partners. Emotional volatility amplifies these dynamics, as rapid mood shifts or irritability often provoke tension and misunderstanding [201].

Empirical evidence confirms that greater ADHD symptom severity predicts poorer interpersonal adjustment and higher rates of relational instability. Couples in which one partner has ADHD report significantly more conflict, poorer communication, and less adaptive conflict-resolution strategies compared to control couples [201]. Family systems are similarly affected: adults with ADHD frequently struggle to meet domestic obligations such as managing finances or maintaining household organization, contributing to family chaos and stress [202,203]. Parenting poses a distinct challenge, as parents with ADHD are more likely to demonstrate inconsistent discipline and reduced involvement, both of which undermine family cohesion and child behavioral adjustment [203]. These findings suggest that the interpersonal sequelae of ADHD reflect not only cognitive deficits but also the cascading effects of emotional and self-regulatory impairments on daily life.

Low self-esteem and social anxiety further compound relational difficulties. Many adults with ADHD internalize years of negative feedback and social rejection, leading to persistent feelings of inadequacy [201]. Quantitative studies demonstrate significantly lower self-esteem and higher divorce rates in this population [14,202]. Social anxiety, affecting up to half of adults with ADHD [204], frequently co-occurs and exacerbates avoidance behaviors, reinforcing isolation and impairing the formation of supportive networks. This combination of low self-worth, emotional lability, and avoidance creates a self-perpetuating cycle that undermines social functioning.

Nonetheless, supportive interpersonal environments can substantially buffer these effects. Evidence indicates that understanding, structured, and nonjudgmental relationships enhance coping and reduce functional impairment [202,205]. Partners who assist with organization and provide consistent emotional support often help mitigate the everyday consequences of ADHD, highlighting the importance of psychoeducation for spouses and family members. Guidelines such as NICE NG87 explicitly recommend addressing relational difficulties in clinical management, emphasizing social support as a core therapeutic target [116].

Sexual and emotional intimacy are also affected. Adults with ADHD report lower sexual satisfaction and overall relationship fulfillment compared to controls [206], with partners of individuals with ADHD describing similar dissatisfaction. Distractibility, forgetfulness, and emotional reactivity can erode intimacy and trust, while chronic disorganization often translates into missed commitments and unbalanced household responsibilities [201,203]. Parenting studies reveal that ADHD symptoms in mothers and fathers contribute to household chaos and inconsistent routines, which in turn heighten family stress and conflict. When both partners have ADHD, these effects are magnified, leading to especially high rates of discord and negative conflict styles [201,203].

Overall, the literature portrays adult ADHD as a condition with profound social and familial ramifications rooted in executive and emotional dysregulation. However, these outcomes are modifiable. Interventions that target emotion regulation, communication skills, and family psychoeducation show promise in enhancing relational satisfaction and stability. Integrating such approaches into standard ADHD care can promote not only symptom management but also improved interpersonal functioning and quality of life.

## 8. Future Research Directions

Despite numerous studies on ADHD in adults, many aspects of the condition still require a deeper understanding, particularly in the context of its etiology, neurobiology, and diagnostic methods. Current treatments and diagnostic tools have their limitations; therefore, future research must focus on several key areas that may contribute to more accurate identification and more effective treatment of adult ADHD.

### 8.1. Better Understanding of Etiology and Neurobiology

The etiology of adult ADHD is complex and multifactorial, involving interactions among genetic, neurobiological, and environmental factors. Recent advances in genetics have enabled the identification of numerous genetic polymorphisms associated with ADHD, including variants in genes encoding DA transporters (DAT1), DA receptor D4 (DRD4), as well as genes related to NA and serotonin metabolism [57]. However, many of these studies are limited to pediatric populations, and evidence concerning adults remains fragmented. Future research should include large, heterogeneous cohorts of adult patients to identify specific genetic variants and their impact on the course of the disorder later in life.

At the same time, both functional and structural neuroimaging studies provide important insights into dysfunctions in brain networks involved in attention, impulsivity, and cognitive control—particularly within the prefrontal cortex, striatum, and limbic system [207]. Advanced techniques such as fMRI, PET, and diffusion tensor imaging allow for detailed analysis of neuronal connectivity and neurotransmitter activity in adults with ADHD. Nevertheless, most current studies are cross-sectional and involve limited sample sizes. Future research projects should emphasize long-term, multicenter prospective studies that allow for tracking neurobiological changes over time and their correlation with clinical symptoms and treatment response [208].

Equally important is the identification of biomarkers for ADHD that could serve as objective diagnostic and prognostic indicators. Potential biomarkers include neurochemical parameters (e.g., neurotransmitter and metabolite levels), inflammatory and oxidative stress markers, as well as metabolomic and epigenetic profiles. Existing studies have shown elevated levels of proinflammatory cytokines and impaired antioxidant function in individuals with ADHD [209], yet their specificity and sensitivity remain insufficient. Integrating biological data with clinical and neuroimaging findings using a multi-omics approach may lead to breakthroughs in personalized diagnosis and treatment.

The influence of environmental and epigenetic factors that modulate gene expression and may determine the ADHD phenotype should not be overlooked. Future studies should investigate the impact of environmental toxin exposure, prenatal stress, nutritional deficiencies, and psychosocial factors on the development and progression of ADHD in adulthood [210]. A deeper understanding of epigenetic mechanisms could pave the way for novel therapeutic interventions aimed at modifying gene expression and restoring neurobiological balance.

### 8.2. Improvement of Diagnostic Methods

The diagnosis of ADHD in adults still relies predominantly on subjective tools, such as clinical interviews and standardized self-report or observer-report questionnaires (e.g., the Conners’ Adult ADHD Rating Scale, ASRS). While widely used, these methods often fail to capture the variability of symptom presentation in adults and the frequent presence of comorbid psychiatric conditions such as depression, anxiety disorders, or substance use disorders, which can complicate accurate diagnosis and treatment planning [4].

In response to these challenges, there is growing interest in developing more objective and precise diagnostic tools. Promising approaches include the use of neuropsychological tests supported by artificial intelligence, which can analyze patterns of attention, impulsivity, and cognitive control with high sensitivity and specificity. Additionally, advances in digital technologies, including mobile applications that monitor patients’ daily functioning and remote symptom assessment systems, enable the collection and analysis of real-time data in naturalistic settings. Telemedicine also improves access to specialized diagnostics, especially in regions with limited availability of psychiatric care.

Another important direction involves improving differential diagnosis to distinguish ADHD from other psychiatric disorders with overlapping symptoms and to identify comorbid conditions. To achieve this, the development of diagnostic protocols that incorporate biomarkers, neuroimaging, and advanced psychometric tools has been proposed, which could significantly enhance diagnostic accuracy.

Moreover, research on the heterogeneity of adult ADHD should aim to identify clinical and biological subtypes, thereby enabling personalized treatment approaches. Evidence suggests that different ADHD phenotypes are associated with distinct neurochemical, genetic, and treatment response profiles—both pharmacological and non-pharmacological. A better understanding of these differences may facilitate the development of targeted interventions and improve treatment effectiveness.

In summary, future research on adult ADHD should integrate genetic, neurobiological, and technological approaches to develop precise, objective, and accessible diagnostic methods as well as tailored therapeutic strategies. Interdisciplinary collaboration among clinicians, neurobiologists, geneticists, and digital technology specialists will be crucial to advancing the field.

### 8.3. Trends and Future Directions in Pharmacotherapy for Adult ADHD

Pharmacotherapy remains one of the core pillars in the treatment of ADHD in adults; however, many aspects of optimizing treatment strategies require further investigation. Currently, the most frequently prescribed medications include psychostimulants, primarily methylphenidate and amphetamines, as well as non-stimulant alternatives such as atomoxetine and guanfacine. Although the efficacy of these agents is well established, treatment response varies considerably among individuals, and tolerability as well as the profile of adverse effects often limit their long-term use.

Future research should focus on identifying predictive factors of individual response to specific medications to facilitate personalized pharmacotherapy. Pharmacogenetic studies, which examine how genetic polymorphisms influence drug metabolism, therapeutic efficacy, and the risk of side effects, represent a promising direction. For example, variants in genes coding for drug-metabolizing enzymes (e.g., CYP450) or neurotransmitter receptors (e.g., dopaminergic, noradrenergic) may determine individual sensitivity to pharmacological treatment and drug tolerability.

Moreover, there is a pressing need for the development of novel compounds with innovative mechanisms of action that could offer more effective and safer symptom control. Research on agents targeting the glutamatergic system, neuroactive peptides, or the endocannabinoid system opens new therapeutic avenues. Equally important is the exploration of combination pharmacotherapies and the development of treatment protocols that adjust dosage and administration schedules to individual patient profiles.

Another critical area is the assessment of long-term safety and efficacy of ADHD medications in adults, especially in the context of psychiatric comorbidities and their impact on quality of life, social functioning, and occupational performance. Large-scale, multicenter randomized controlled trials with extended follow-up periods are essential to evaluate the real-world benefits and risks of pharmacological treatment.

Furthermore, pharmacotherapy should be integrated with non-pharmacological interventions such as psychotherapy, dietary interventions, and neurofeedback. Combined treatment approaches may lead to superior therapeutic outcomes. Research into optimal models for integrated care and the appropriate timing for initiating specific modalities is necessary to maximize treatment efficacy.

In conclusion, future pharmacological research in adult ADHD should prioritize the personalization of therapy, the development of innovative pharmacological agents, and the evaluation of long-term treatment outcomes. A holistic, interdisciplinary approach will be essential to advancing clinical practice and improving the quality of life for adults living with ADHD.

## 9. Limitations

This narrative review provides a broad synthesis of current evidence on adult ADHD; certain limitations should be noted. First, the absence of a formal meta-analytic approach means that effect sizes and quantitative comparisons between studies cannot be established. Nonetheless, a narrative methodology was chosen deliberately to allow a more flexible and integrative discussion across heterogeneous study designs and research domains. Second, variability in diagnostic criteria, assessment tools, and sample characteristics across the included studies introduces heterogeneity, which reflects the current state of the field and underscores the need for greater standardization in future research. Third, much of the available literature relies on retrospective self-reports to establish childhood symptom onset, which may be subject to recall bias. Finally, although the search strategy was comprehensive and included multiple databases, the possibility of publication bias and the exclusion of very recent publications cannot be fully ruled out. Despite these constraints, this review highlights consistent patterns across studies, identifies critical gaps in knowledge, and outlines priorities that can guide future research and clinical practice.

## 10. Conclusions

Adult ADHD is a complex and heterogeneous disorder with far-reaching implications for individual functioning and public health. The current evidence underscores the importance of recognizing its distinct clinical features in adulthood, the need for reliable diagnostic tools, and the effectiveness of integrated treatment approaches that combine pharmacotherapy with psychosocial interventions. Despite advances in understanding, significant knowledge gaps remain, particularly regarding neurobiological markers and individualized treatment strategies. Addressing these gaps through robust, multidisciplinary research will be essential to improve early identification, optimize therapeutic outcomes, and ultimately enhance quality of life for adults living with ADHD.

## Figures and Tables

**Table 1:** Estimated prevalence of ADHD in adults across different regions.

Region	Estimated Prevalence (%)	Methodological and Clinical Interpretation	Source
**Globally**	2.5–6.7% (Average approx. 2.8%)	Very large variability; lower values (~2.5%) reflect strict clinical diagnoses, higher values (up to 6.7%) come from screening studies based on self-report.	[2,5]
**Europe (EU)**	2.0–5.0% (Typically 3–4%)	Likely underestimation due to differences in access to diagnosis and variation in healthcare systems.	[5,24,25]
**North America (USA)**	~4.4%	Higher rate than in Europe, likely reflecting greater clinical awareness and more developed diagnostic infrastructure.	[26,27,28]
**North America (Canada)**	~3.3%	Heterogeneous results, similar to European estimates.	[29,30]
**South America**	2.0–10.0%	Limited and highly inconsistent methodological data; reliable conclusions cannot be drawn.	[5]
**Asia**	2.0–6.9%	High variability; reporting strongly influenced by cultural factors, including mental health stigma.	[31,32]
**Middle East and North Africa**	~13.5%	Rate appears extremely high compared to other regions; requires urgent methodological verification.	[24]
**Australia**	~3.4%	Consistent with other high-income countries with developed healthcare systems (Europe, Canada).	[33]

**Table 2:** Selected epidemiological studies on the first ADHD diagnosis in adulthood (age ≥ 18 years).

Region	Sample (Type)	Age	Key Finding: % First Diagnosed in Adulthood	Source
**USA (national)**	Population survey (NCHS RSS, N ≈ 7000)	≥18 years	55.9% of adults with ADHD were first diagnosed at age ≥ 18	[35]
**USA**	Psychiatrists/PCPs chart review (N = 854)	≥18 years	~75% were first diagnosed in adulthood (only 25% had a childhood diagnosis)	[36]
**Japan**	National insurance claims (N = 838,265 new ADHD)	≥20 years	40% of all new ADHD cases were in adults	[37]

**Table 4:** Functional domains, DSM-5 symptom examples, and associated clinical features in ADHD.

Domain	Functional Component	DSM-5 Symptom Examples	Common Clinical Features and Underlying Deficits	Source
**Cognitive (Inattention)**	Sustained Attention	“Fails to give close attention to details”	Difficulty maintaining focus during monotonous tasks (e.g., lectures, reading); high susceptibility to distraction	[87,91]
**Executive Function (Working Memory)**	Working Memory Capacity	“Often forgetful in daily activities”	Forgets multi-step directions; difficulty holding and manipulating information (e.g., mental math, instructions)	[87,91]
**Executive Function (Organization)**	Task Planning and Organization	“Difficulty organizing tasks and activities”	Poor time management; chronic lateness; disorganized environment; difficulty initiating tasks (procrastination).	[87,91]
**Cognitive (Hyperactivity/Impulsivity)**	Behavioral Inhibitory Control	“Often interrupts or intrudes on others”	Blurting out answers; hasty decisions without adequate information; difficulty waiting in line.	[92,93]
**Hyperactivity (Internalized)**	Internal Restlessness	“Is often ‘on the go’ acting as if ‘driven by a motor’”	Subjective inner restlessness; difficulty relaxing; fidgeting (e.g., tapping pen, shaking leg).	[92,94]
**Emotional (Non-DSM Core Criteria)**	Emotional Instability/Dysregulation	Not listed as core DSM-5 symptom (classified as “associated feature”)	Low frustration tolerance; rapid, intense mood swings; irritability; difficulty calming down.	[95]
**Emotional Impulsivity**	Emotional Urgency/Affective Impulsivity	Not listed as core DSM-5 symptom	“Negative urgency”; rash actions under emotional stress (e.g., angry emails, impulsive purchases).	[95]

## Data Availability

No new data were created or analyzed in this study. Data sharing is not applicable to this article.

## References

[1] Hrys A., Chepeleva N., Tkachuk T., Tor L. (2024). Aдaптaцiйний пoтeнцiaл ociб дopocлoгo вiкy з poзлaдoм дeфiцитy yвaги/гiпepaктивнocтi (PДУГ). Insight Psychol. Dimens. Soc..

[2] Al-Yateem N., Slewa-Younan S., Halimi A., Saeed S.A., Tliti D., Mohammad M., Ridwan M., Zeidan R., Hammash M.H., Ahmed F.R. (2023). Prevalence of Undiagnosed Attention Deficit Hyperactivity Disorder (ADHD) Symptoms in the Young Adult Population of the United Arab Emirates: A National Cross-Sectional Study. J. Epidemiol. Glob. Health.

[3] Johnson J., Morris S., George S. (2020). Attention deficit hyperactivity disorder in adults: What the non-specialist needs to know. Br. J. Hosp. Med..

[4] Kooij J., Bijlenga D., Salerno L., Jaeschke R., Bitter I., Balázs J., Thome J., Dom G., Kasper S., Filipe C.N. (2019). Updated European Consensus Statement on diagnosis and treatment of adult ADHD. Eur. Psychiatry.

[5] Song P., Zha M., Yang Q., Zhang Y., Li X., Rudan I. (2021). The prevalence of adult attention-deficit hyperactivity disorder: A global systematic review and meta-analysis. J. Glob. Health.

[6] Taylor M.J., Larsson H., Gillberg C., Lichtenstein P., Lundström S. (2019). Investigating the childhood symptom profile of community-based individuals diagnosed with attention-deficit/hyperactivity disorder as adults. J. Child Psychol. Psychiatry.

[7] Breda V., Rovaris D.L., Vitola E.S., Mota N.R., Blaya-Rocha P., Salgado C.A.I., Victor M.M., Picon F.A., Karam R.G., Silva K.L. (2016). Does collateral retrospective information about childhood attention-deficit/hyperactivity disorder symptoms assist in the diagnosis of attention-deficit/hyperactivity disorder in adults? Findings from a large clinical sample. Aust. N. Zeal. J. Psychiatry.

[8] Lin Y.-J., Yang L.-K., Gau S.S.-F. (2016). Psychiatric comorbidities of adults with early- and late-onset attention-deficit/hyperactivity disorder. Aust. N. Zeal. J. Psychiatry.

[9] Liu C.-Y., Asherson P., Viding E., Greven C.U., Pingault J.-B. (2021). Early Predictors of De Novo and Subthreshold Late-Onset ADHD in a Child and Adolescent Cohort. J. Atten. Disord..

[10] Turjeman-Levi Y., Itzchakov G., Engel-Yeger B. (2024). Executive function deficits mediate the relationship between employees’ ADHD and job burnout. AIMS Public Health.

[11] Lauder K., McDowall A., Tenenbaum H.R. (2022). A systematic review of interventions to support adults with ADHD at work—Implications from the paucity of context-specific research for theory and practice. Front. Psychol..

[12] Soares L.S., Alves A.L.C., Costa D.d.S., Malloy-Diniz L.F., de Paula J.J., Romano-Silva M.A., de Miranda D.M. (2021). Common Venues in Romantic Relationships of Adults With Symptoms of Autism and Attention Deficit/Hyperactivity Disorder. Front. Psychiatry.

[13] Oguchi M., Takahashi T., Nitta Y., Kumano H. (2021). The Moderating Effect of Attention-Deficit Hyperactivity Disorder Symptoms on the Relationship Between Procrastination and Internalizing Symptoms in the General Adult Population. Front. Psychol..

[14] Katzman M.A., Bilkey T.S., Chokka P.R., Fallu A., Klassen L.J. (2017). Adult ADHD and comorbid disorders: Clinical implications of a dimensional approach. BMC Psychiatry.

[15] Chen Q., A Hartman C., Haavik J., Harro J., Klungsøyr K., Hegvik T.-A., Wanders R., Ottosen C., Dalsgaard S., Faraone S.V. (2018). Common psychiatric and metabolic comorbidity of adult attention-deficit/hyperactivity disorder: A population-based cross-sectional study. PLoS ONE.

[16] van de Glind G., Konstenius M., Koeter M.W.J., van Emmerik-van Oortmerssen K., Carpentier P.-J., Kaye S., Degenhardt L., Skutle A., Franck J., Bu E.-T. (2014). Variability in the prevalence of adult ADHD in treatment seeking substance use disorder patients: Results from an international multi-center study exploring DSM-IV and DSM-5 criteria. Drug Alcohol Depend..

[17] Weibel S., Bicego F., Muller S., Martz E., Costache M.E., Kraemer C., Bertschy G., Lopez R., Weiner L. (2022). Two Facets of Emotion Dysregulation Are Core Symptomatic Domains in Adult ADHD: Results from the SR-WRAADDS, a Broad Symptom Self-Report Questionnaire. J. Atten. Disord..

[18] Green J.G., DeYoung G., Wogan M.E., Wolf E.J., Lane K.L., Adler L.A. (2018). Evidence for the reliability and preliminary validity of the Adult ADHD Self-Report Scale v1.1 (ASRS v1.1) Screener in an adolescent community sample. Int. J. Methods Psychiatr. Res..

[19] Cohen M.B.-D., Eldar E., Maeir A., Nahum M. (2021). Emotional dysregulation and health related quality of life in young adults with ADHD: A cross sectional study. Health Qual. Life Outcomes.

[20] Widding-Havneraas T., Markussen S., Elwert F., Lyhmann I., Bjelland I., Halmøy A., Chaulagain A., Ystrom E., Mykletun A., Zachrisson H.D. (2023). Geographical variation in ADHD: Do diagnoses reflect symptom levels?. Eur. Child Adolesc. Psychiatry.

[21] Park S., Park S. (2024). Prevalence, Correlates, and Comorbidities Among Young Adults Who Screened Positive for ADHD in South Korea During the COVID-19 Pandemic. J. Atten. Disord..

[22] Stickley A., Leinsalu M., Ruchkin V., Oh H., Narita Z., Koyanagi A. (2019). Attention-deficit/hyperactivity disorder symptoms and perceived mental health discrimination in adults in the general population. Eur. Psychiatry.

[23] Vater C.H., DiSalvo M., Ehrlich A., Parker H., O’cOnnor H., Faraone S.V., Biederman J. (2024). ADHD in Adults: Does Age at Diagnosis Matter?. J. Atten. Disord..

[24] Al-Wardat M., Etoom M., A Almhdawi K., Hawamdeh Z., Khader Y. (2024). Prevalence of attention-deficit hyperactivity disorder in children, adolescents and adults in the Middle East and North Africa region: A systematic review and meta-analysis. BMJ Open.

[25] Caye A., Swanson J., Thapar A., Sibley M., Arseneault L., Hechtman L., Arnold L.E., Niclasen J., Moffitt T., Rohde L.A. (2016). Life Span Studies of ADHD—Conceptual Challenges and Predictors of Persistence and Outcome. Curr. Psychiatry Rep..

[26] Morkem R., Handelman K., Queenan J.A., Birtwhistle R., Barber D. (2020). Validation of an EMR algorithm to measure the prevalence of ADHD in the Canadian Primary Care Sentinel Surveillance Network (CPCSSN). BMC Med. Inform. Decis. Mak..

[27] Kessler R.C., Adler L., Barkley R., Biederman J., Conners C.K., Demler O., Faraone S.V., Greenhill L.L., Howes M.J., Secnik K. (2006). The prevalence and correlates of adult ADHD in the United States: Results from the National Comorbidity Survey Replication. Am. J. Psychiatry.

[28] Wilens T.E., Martelon M., Joshi G., Bateman C., Fried R., Petty C., Biederman J. (2011). Does ADHD Predict Substance-Use Disorders? A 10-Year Follow-up Study of Young Adults With ADHD. J. Am. Acad. Child Adolesc. Psychiatry.

[29] Vingilis E., Erickson P.G., Toplak M.E., Kolla N.J., Mann R.E., Seeley J., Vandermaas M., Daigle D.S. (2015). Attention Deficit Hyperactivity Disorder Symptoms, Comorbidities, Substance Use, and Social Outcomes among Men and Women in a Canadian Sample. BioMed Res. Int..

[30] Espinet S.D., Graziosi G., Toplak M.E., Hesson J., Minhas P. (2022). A Review of Canadian Diagnosed ADHD Prevalence and Incidence Estimates Published in the Past Decade. Brain Sci..

[31] van de Glind G., van den Brink W., Koeter M.W., Carpentier P.J., van Emmerik-van Oortmerssen K., Kaye S., Skutle A., Bu E.-T.H., Franck J., Konstenius M. (2013). Validity of the Adult ADHD Self-Report Scale (ASRS) as a screener for adult ADHD in treatment seeking substance use disorder patients. Drug Alcohol Depend..

[32] Slobodin O., Crunelle C.L. (2019). Mini Review: Socio-Cultural Influences on the Link Between ADHD and SUD. Front. Public Health.

[33] Ebejer J.L., E Medland S., Van Der Werf J., Gondro C., Henders A.K., Lynskey M., Martin N.G., Duffy D.L. (2012). Attention deficit hyperactivity disorder in australian adults: Prevalence, persistence, conduct problems and disadvantage. PLoS ONE.

[34] Caye A., Rocha T.B.-M., Anselmi L., Murray J., Menezes A.M.B., Barros F.C., Gonçalves H., Wehrmeister F., Jensen C.M., Steinhausen H.-C. (2016). Attention-Deficit/Hyperactivity Disorder Trajectories From Childhood to Young Adulthood: Evidence From a Birth Cohort Supporting a Late-Onset Syndrome. JAMA Psychiatry.

[35] Staley B.S., Robinson L.R., Claussen A.H., Katz S.M., Danielson M.L., Summers A.D., Farr S.L., Blumberg S.J., Tinker S.C. (2024). Attention-Deficit/Hyperactivity Disorder Diagnosis, Treatment, and Telehealth Use in Adults—National Center for Health Statistics Rapid Surveys System, United States, October–November 2023. MMWR Morb. Mortal Wkly. Rep..

[36] Faraone S.V., Spencer T.J., Montano C.B., Biederman J. (2004). Attention-deficit/hyperactivity disorder in adults: A survey of current practice in psychiatry and primary care. Arch. Intern Med..

[37] Sasayama D., Kuge R., Toibana Y., Honda H. (2022). Trends in Diagnosed Attention-Deficit/Hyperactivity Disorder Among Children, Adolescents, and Adults in Japan From April 2010 to March 2020. JAMA Netw. Open.

[38] Brod M., Pohlman B., Lasser R., Hodgkins P. (2012). Comparison of the burden of illness for adults with ADHD across seven countries: A qualitative study. Health Qual. Life Outcomes.

[39] Mattos P., Louzã M.R., Palmini A.L.F., de Oliveira I.R., Rocha F.L. (2013). A multicenter, open-label trial to evaluate the quality of life in adults with ADHD treated with long-acting methylphenidate (OROS MPH): Concerta Quality of Life (CONQoL) study. J. Atten. Disord..

[40] Hesson J., Fowler K. (2018). Prevalence and Correlates of Self-Reported ADD/ADHD in a Large National Sample of Canadian Adults. J. Atten. Disord..

[41] Mao A.R., Findling R.L. (2014). Comorbidities in adult attention-deficit/hyperactivity disorder: A practical guide to diagnosis in primary care. Postgrad. Med..

[42] Zaleski A.L., Craig K.J.T., Khan R., Waber R., Xin W., Powers M., Ramey U., Verbrugge D.J., Fernandez-Turner D. (2025). Real-world evaluation of prevalence, cohort characteristics, and healthcare utilization and expenditures among adults and children with autism spectrum disorder, attention-deficit hyperactivity disorder, or both. BMC Health Serv. Res..

[43] Lai M.-C., Kassee C., Besney R., Bonato S., Hull L., Mandy W., Szatmari P., Ameis S.H. (2019). Prevalence of co-occurring mental health diagnoses in the autism population: A systematic review and meta-analysis. Lancet Psychiatry.

[44] Martinez S., Stoyanov K., Carcache L. (2024). Unraveling the spectrum: Overlap, distinctions, and nuances of ADHD and ASD in children. Front. Psychiatry.

[45] Harikumar A., Evans D.W., Dougherty C.C., Carpenter K.L., Michael A.M. (2021). A Review of the Default Mode Network in Autism Spectrum Disorders and Attention Deficit Hyperactivity Disorder. Brain Connect..

[46] Rodenas-Cuadrado P., Ho J., Vernes S.C. (2014). Shining a light on CNTNAP2: Complex functions to complex disorders. Eur. J. Hum. Genet..

[47] E Yerys B., A McQuaid G., Lee N.R., Wallace G.L. (2022). Co-occurring ADHD symptoms in autistic adults are associated with less independence in daily living activities and lower subjective quality of life. Autism.

[48] Deserno M.K., Bathelt J., Groenman A.P., Geurts H.M. (2023). Probing the overarching continuum theory: Data-driven phenotypic clustering of children with ASD or ADHD. Eur. Child Adolesc. Psychiatry.

[49] Schweizer T., Endres D., Dziobek I., van Elst L.T. (2024). Psychosocial therapeutic approaches for high-functioning autistic adults. Front. Psychiatry.

[50] Young S., Hollingdale J., Absoud M., Bolton P., Branney P., Colley W., Craze E., Dave M., Deeley Q., Farrag E. (2020). Guidance for identification and treatment of individuals with attention deficit/hyperactivity disorder and autism spectrum disorder based upon expert consensus. BMC Med..

[51] Yoshimasu K., Barbaresi W.J., Colligan R.C., Voigt R.G., Killian J.M., Weaver A.L., Katusic S.K. (2018). Adults With Persistent ADHD: Gender and Psychiatric Comorbidities—A Population-Based Longitudinal Study. J. Atten. Disord..

[52] Instanes J.T., Klungsøyr K., Halmøy A., Fasmer O.B., Haavik J. (2018). Adult ADHD and Comorbid Somatic Disease: A Systematic Literature Review. J. Atten. Disord..

[53] Surman C.B., Monuteaux M.C., Petty C.R., Faraone S.V., Spencer T.J., Chu N.F., Biederman J. (2010). Representativeness of participants in a clinical trial for attention-deficit/hyperactivity disorder? Comparison with adults from a large observational study. J. Clin. Psychiatry.

[54] Kooij J.J.S., Huss M., Asherson P., Akehurst R., Beusterien K., French A., Sasané R., Hodgkins P. (2012). Distinguishing comorbidity and successful management of adult ADHD. J. Atten. Disord..

[55] Okada T., Sotodate T., Ogasawara-Shimizu M., Nishigaki N. (2024). Psychiatric comorbidities of attention deficit/hyperactivity disorder in Japan: A nationwide population-based study. Front. Psychiatry.

[56] Kofler M.J., Singh L.J., Soto E.F., Chan E.S.M., Miller C.E., Harmon S.L., Spiegel J.A. (2020). Working memory and short-term memory deficits in ADHD: A bifactor modeling approach. Neuropsychology.

[57] Faraone S.V., Larsson H. (2019). Genetics of attention deficit hyperactivity disorder. Mol. Psychiatry.

[58] Faraone S.V., Banaschewski T., Coghill D., Zheng Y., Biederman J., Bellgrove M.A., Newcorn J.H., Gignac M., Al Saud N.M., Manor I. (2021). The World Federation of ADHD International Consensus Statement: 208 Evidence-based conclusions about the disorder. Neurosci. Biobehav. Rev..

[59] Demontis D., Walters R.K., Martin J., Mattheisen M., Als T.D., Agerbo E., Baldursson G., Belliveau R., Bybjerg-Grauholm J., Bækvad-Hansen M. (2019). Discovery of the first genome-wide significant risk loci for attention deficit/hyperactivity disorder. Nat. Genet..

[60] Grünblatt E., Werling A.M., Roth A., Romanos M., Walitza S. (2019). Association study and a systematic meta-analysis of the VNTR polymorphism in the 3′-UTR of dopamine transporter gene and attention-deficit hyperactivity disorder. J. Neural Transm..

[61] Acosta H., Kantojärvi K., Tuulari J.J., Lewis J.D., Hashempour N., Scheinin N.M., Lehtola S.J., Nolvi S., Fonov V.S., Collins D.L. (2023). Association of cumulative prenatal adversity with infant subcortical structure volumes and child problem behavior and its moderation by a coexpression polygenic risk score of the serotonin system. Dev. Psychopathol..

[62] Ni M., Li L., Li W., Zhang Q., Zhao J., Shen Q., Yao D., Wang T., Li B., Ding X. (2023). Examining the relationship between birth weight and attention-deficit hyperactivity disorder diagnosis. Front. Psychiatry.

[63] Sánchez-Mora C., Richarte V., Garcia-Martínez I., Pagerols M., Corrales M., Bosch R., Vidal R., Viladevall L., Casas M., Cormand B. (2015). Dopamine receptor DRD4 gene and stressful life events in persistent attention deficit hyperactivity disorder. Am. J. Med. Genet. Part B Neuropsychiatr. Genet..

[64] Todd R.D., Neuman R.J. (2007). Gene–Environment interactions in the development of combined type ADHD: Evidence for a synapse-based model. Am. J. Med. Genet. Part B Neuropsychiatr. Genet..

[65] Lundström S., Chang Z., Kerekes N., Gumpert C.H., Råstam M., Gillberg C., Lichtenstein P., Anckarsäter H. (2011). Autistic-like traits and their association with mental health problems in two nationwide twin cohorts of children and adults. Psychol. Med..

[66] Fusar-Poli P., Rubia K., Rossi G., Sartori G., Balottin U. (2012). Striatal dopamine transporter alterations in ADHD: Pathophysiology or adaptation to psychostimulants? A meta-analysis. Am. J. Psychiatry.

[67] Véronneau-Veilleux F., Robaey P., Ursino M., Nekka F. (2022). A mechanistic model of ADHD as resulting from dopamine phasic/tonic imbalance during reinforcement learning. Front. Comput. Neurosci..

[68] Van Dessel J., Sonuga-Barke E., Mies G., Lemiere J., Van der Oord S., Morsink S., Danckaerts M. (2018). Delay aversion in attention deficit/hyperactivity disorder is mediated by amygdala and prefrontal cortex hyper-activation. J. Child Psychol. Psychiatry.

[69] Gamo N.J., Wang M., Arnsten A.F. (2010). Methylphenidate and atomoxetine enhance prefrontal function through α2-adrenergic and dopamine D1 receptors. J. Am. Acad. Child Adolesc. Psychiatry.

[70] Vidor M.V., Panzenhagen A.C., Martins A.R., Cupertino R.B., Bandeira C.E., Picon F.A., da Silva B.S., Vitola E.S., Rohde L.A., Rovaris D.L. (2022). Emerging findings of glutamate–glutamine imbalance in the medial prefrontal cortex in attention deficit/hyperactivity disorder: Systematic review and meta-analysis of spectroscopy studies. Eur. Arch. Psychiatry Clin. Neurosci..

[71] Faraone S.V., Ward C.L., Boucher M., Elbekai R., Brunner E. (2025). Role of serotonin in psychiatric and somatic comorbidities of attention-deficit/hyperactivity disorder: A systematic literature review. Neurosci. Biobehav. Rev..

[72] Hoogman M., Muetzel R., Guimaraes J.P., Shumskaya E., Mennes M., Zwiers M.P., Jahanshad N., Sudre G., Wolfers T., Earl E.A. (2019). Brain Imaging of the Cortex in ADHD: A Coordinated Analysis of Large-Scale Clinical and Population-Based Samples. Am. J. Psychiatry.

[73] Shaw P., Eckstrand K., Sharp W., Blumenthal J., Lerch J.P., Greenstein D., Clasen L., Evans A., Giedd J., Rapoport J.L. (2007). Attention-deficit/hyperactivity disorder is characterized by a delay in cortical maturation. Proc. Natl. Acad. Sci. USA.

[74] van Ewijk H., Heslenfeld D.J., Zwiers M.P., Buitelaar J.K., Oosterlaan J. (2012). Diffusion tensor imaging in attention deficit/hyperactivity disorder: A systematic review and meta-analysis. Neurosci. Biobehav. Rev..

[75] Hamilton L.S., Levitt J.G., O’Neill J., Alger J.R., Luders E., Phillips O.R., Caplan R., Toga A.W., McCracken J., Narr K.L. (2008). Reduced white matter integrity in attention-deficit hyperactivity disorder. Neuroreport.

[76] Stephan K.E., Friston K.J. (2010). Analyzing effective connectivity with functional magnetic resonance imaging. WIREs Cogn. Sci..

[77] Stoodley C.J. (2016). The Cerebellum and Neurodevelopmental Disorders. Cerebellum.

[78] Hilger K., Fiebach C.J. (2019). ADHD symptoms are associated with the modular structure of intrinsic brain networks in a representative sample of healthy adults. Netw. Neurosci..

[79] Castellanos F.X., Proal E. (2012). Large-scale brain systems in ADHD: Beyond the prefrontal–striatal model. Trends Cogn. Sci..

[80] Lin H.-Y., Tseng W.-Y.I., Lai M.-C., Matsuo K., Gau S.S.-F. (2015). Altered Resting-State Frontoparietal Control Network in Children with Attention-Deficit/Hyperactivity Disorder. J. Int. Neuropsychol. Soc..

[81] Vera J.D., Freichel R., Michelini G., Loo S.K., Lenartowicz A. (2024). A Network Approach to Understanding the Role of Executive Functioning and Alpha Oscillations in Inattention and Hyperactivity-Impulsivity Symptoms of ADHD. J. Atten. Disord..

[82] Ishii-Takahashi A., Takizawa R., Nishimura Y., Kawakubo Y., Kuwabara H., Matsubayashi J., Hamada K., Okuhata S., Yahata N., Igarashi T. (2013). Prefrontal activation during inhibitory control measured by near-infrared spectroscopy for differentiating between autism spectrum disorders and attention deficit hyperactivity disorder in adults. Neuroimage Clin..

[83] Sáenz A.A., Villemonteix T., Massat I. (2019). Structural and functional neuroimaging in attention-deficit/hyperactivity disorder. Dev. Med. Child Neurol..

[84] Salomone S., Fleming G.R., Bramham J., O’cOnnell R.G., Robertson I.H. (2020). Neuropsychological Deficits in Adult ADHD: Evidence for Differential Attentional Impairments, Deficient Executive Functions, and High Self-Reported Functional Impairments. J. Atten. Disord..

[85] Dunn G.A., Nigg J.T., Sullivan E.L. (2019). Neuroinflammation as a risk factor for attention deficit hyperactivity disorder. Pharmacol. Biochem. Behav..

[86] Lewis N., Villani A., Lagopoulos J. (2025). Gut dysbiosis as a driver of neuroinflammation in attention-deficit/hyperactivity disorder: A review of current evidence. Neuroscience.

[87] DSM. https://www.psychiatry.org:443/psychiatrists/practice/dsm.

[88] Posner J., Polanczyk G.V., Sonuga-Barke E. (2020). Attention-deficit hyperactivity disorder. Lancet.

[89] Bedawi R.M., Al-Farsi Y., Mirza H., Al-Huseini S., Al-Mahrouqi T., Al-Kiyumi O., Al-Azri M., Al-Adawi S. (2024). Prevalence and Clinical Profile of Adults with ADHD Attending a Tertiary Care Hospital for Five Years. Int. J. Environ. Res. Public Health.

[90] LeRoy A., Jacova C., Young C. (2019). Neuropsychological Performance Patterns of Adult ADHD Subtypes. J. Atten. Disord..

[91] Hackett A.P.-C., Joseph R.P.-C., Robinson K.P.-C., Welsh J.D., Nicholas J., Schmidt E. (2020). Adult attention deficit/hyperactivity disorder in the ambulatory care setting. J. Am. Acad. Physician Assist..

[92] Mostert J.C., Onnink A.M.H., Klein M., Dammers J., Harneit A., Schulten T., van Hulzen K.J., Kan C.C., Slaats-Willemse D., Buitelaar J.K. (2015). Cognitive heterogeneity in adult attention deficit/hyperactivity disorder: A systematic analysis of neuropsychological measurements. Eur. Neuropsychopharmacol..

[93] Senkowski D., Ziegler T., Singh M., Heinz A., He J., Silk T., Lorenz R.C. (2024). Assessing Inhibitory Control Deficits in Adult ADHD: A Systematic Review and Meta-analysis of the Stop-signal Task. Neuropsychol. Rev..

[94] Rodriguez-Jiménez R., Cubillo A., A Jiménez-Arriero M., Ponce G., Aragüés-Figuero M., Palomo T. (2006). Executive dysfunctions in adults with attention deficit hyperactivity disorder. Rev. Neurol..

[95] Yue X., Liu L., Chen W., Preece D.A., Liu Q., Li H., Wang Y., Qian Q. (2022). Affective-cognitive-behavioral heterogeneity of Attention-Deficit/Hyperactivity Disorder (ADHD): Emotional dysregulation as a sentinel symptom differentiating “ADHD-simplex” and “ADHD-complex” syndromes?. J. Affect. Disord..

[96] Alaghband-Rad J., Dashti B., Tehranidoost M., Zargarinejad G.M., FarhadBeigi P.M. (2021). A Preliminary Investigation of Deficits in Executive Functions of Adults With Attention Deficit Hyperactivity Disorder. J. Nerv. Ment. Dis..

[97] Chan E.S.M., Langberg J.M. (2024). Predicting Occupational Outcomes for Individuals with ADHD: The Role of Hyperactivity/Impulsivity and Executive Functioning. J. Occup. Rehabil..

[98] Guo N., Fuermaier A.B.M., Koerts J., Mueller B.W., Diers K., Mroß A., Mette C., Tucha L., Tucha O. (2021). Neuropsychological functioning of individuals at clinical evaluation of adult ADHD. J. Neural Transm..

[99] Yáñez-Téllez G., Romero-Romero H., Rivera-García L., Prieto-Corona B., Bernal-Hernandez J., Marosi-Holczberger E., Guerrero-Juárez V., Rodríguez-Camacho M., Silva-Pereyra J.F. (2012). Cognitive and executive functions in ADHD. Actas Esp. Psiquiatr..

[100] Holst Y., Thorell L.B. (2017). Neuropsychological Functioning in Adults With ADHD and Adults With Other Psychiatric Disorders. J. Atten. Disord..

[101] Pettersson R., Söderström S., Nilsson K.W. (2018). Diagnosing ADHD in Adults: An Examination of the Discriminative Validity of Neuropsychological Tests and Diagnostic Assessment Instruments. J. Atten. Disord..

[102] de Braek D.I., Dijkstra J.B., Jolles J. (2011). Cognitive Complaints and Neuropsychological Functioning in Adults With and Without Attention-Deficit Hyperactivity Disorder Referred for Multidisciplinary Assessment. Appl. Neuropsychol..

[103] Shaw P., Stringaris A., Nigg J., Leibenluft E. (2014). Emotion Dysregulation in Attention Deficit Hyperactivity Disorder. Am. J. Psychiatry.

[104] Soler-Gutiérrez A.-M., Pérez-González J.-C., Mayas J. (2023). Evidence of emotion dysregulation as a core symptom of adult ADHD: A systematic review. PLoS ONE.

[105] Hirsch O., Chavanon M., Riechmann E., Christiansen H. (2018). Emotional dysregulation is a primary symptom in adult Attention-Deficit/Hyperactivity Disorder (ADHD). J. Affect. Disord..

[106] Fu X., Wu W., Wu Y., Liu X., Liang W., Wu R., Li Y. (2025). Adult ADHD and comorbid anxiety and depressive disorders: A review of etiology and treatment. Front. Psychiatry.

[107] Choi W.-S., Woo Y.S., Wang S.-M., Lim H.K., Bahk W.-M. (2022). The prevalence of psychiatric comorbidities in adult ADHD compared with non-ADHD populations: A systematic literature review. PLoS ONE.

[108] Moldekleiv C.D., Lundervold A.J., Solberg B.S., Engeland A., Fadnes L.T., Chalabianloo F., Klungsøyr K. (2025). Prevalence of substance use disorder in individuals with attention deficit/hyperactivity disorder: Associations with sex and psychiatric comorbidity. BMC Psychiatry.

[109] Shaker N.M., Mahmoud D.A.M., Ahmed M.M., Rabie E.S. (2025). Frequency of binge eating in medication adherent patients with ADHD and its relation to impulsivity. Middle East Curr. Psychiatry.

[110] Hambleton A., Pepin G., Le A., Maloney D., Touyz S., Maguire S. (2022). Psychiatric and medical comorbidities of eating disorders: Findings from a rapid review of the literature. J. Eat. Disord..

[111] Baby M., Priya V., Pallavi J., Chand B.K., Srinivasan L.V., Rana S.S., Tawil S., Haque S., Ghosh P., Bhattacharya P. (2025). A Narrative Review of Outcomes, Comorbidities, and Alternative Behavioral Interventions in Adolescent and Adult Women with ADHD. Int. J. Women’s Health.

[112] Alarachi A., Merrifield C., Rowa K., McCabe R.E. (2024). Are We Measuring ADHD or Anxiety? Examining the Factor Structure and Discriminant Validity of the Adult ADHD Self-Report Scale in an Adult Anxiety Disorder Population. Assessment.

[113] Barbuti M., Maiello M., Spera V., Pallucchini A., Brancati G.E., Maremmani A.G.I., Perugi G., Maremmani I. (2023). Challenges of Treating ADHD with Comorbid Substance Use Disorder: Considerations for the Clinician. J. Clin. Med..

[114] (2018). Overview|Attention Deficit Hyperactivity Disorder: Diagnosis and Management|Guidance|NICE. https://www.nice.org.uk/guidance/ng87.

[115] (2016). Table 7, DSM-IV to DSM-5 Attention-Deficit/Hyperactivity Disorder Comparison. https://www.ncbi.nlm.nih.gov/books/NBK519712/table/ch3.t3/.

[116] (2018). Recommendations|Attention Deficit Hyperactivity Disorder: Diagnosis and Management|Guidance|NICE. https://www.nice.org.uk/guidance/ng87/chapter/Recommendations#diagnosis#.

[117] Ginsberg Y., Quintero J., Anand E., Casillas M., Upadhyaya H.P. (2014). Underdiagnosis of Attention-Deficit/Hyperactivity Disorder in Adult Patients: A Review of the Literature. Prim Care Companion CNS Disord..

[118] Christensen K.S., Storebø O.J., Bach B. (2025). Assessing the Construct Validity of the Adult ADHD Self-report Scale for DSM-5 and Prevalence of ADHD in a Danish Population Sample. J. Atten. Disord..

[119] Brevik E.J., Lundervold A.J., Haavik J., Posserud M. (2020). Validity and accuracy of the Adult Attention-Deficit/Hyperactivity Disorder (ADHD) Self-Report Scale (ASRS) and the Wender Utah Rating Scale (WURS) symptom checklists in discriminating between adults with and without ADHD. Brain Behav..

[120] Hong M., Kooij J.S., Kim B., Joung Y.-S., Yoo H.K., Kim E.-J., Lee S.I., Bhang S.-Y., Lee S.Y., Han D.H. (2020). Validity of the Korean Version of DIVA-5: A Semi-Structured Diagnostic Interview for Adult ADHD. Neuropsychiatr. Dis. Treat..

[121] Lovett B.J., Harrison A.G. (2021). Assessing adult ADHD: New research and perspectives. J. Clin. Exp. Neuropsychol..

[122] Mestres F., Richarte V., Crespín J.J., Torrent C., Biel S., Ramos C., Ibáñez P., Oltra-Arañó L., Corrales M., Amoretti S. (2025). Sex differences in adults with attention-deficit/hyperactivity disorder: A population-based study. Eur. Psychiatry.

[123] Solberg B.S., Halmøy A., Engeland A., Igland J., Haavik J., Klungsøyr K. (2018). Gender differences in psychiatric comorbidity: A population-based study of 40 000 adults with attention deficit hyperactivity disorder. Acta Psychiatr. Scand..

[124] Quinn P.O., Madhoo M. (2014). A review of attention-deficit/hyperactivity disorder in women and girls: Uncovering this hidden diagnosis. Prim Care Companion CNS Disord..

[125] Vildalen V.U., Brevik E.J., Haavik J., Lundervold A.J. (2019). Females With ADHD Report More Severe Symptoms Than Males on the Adult ADHD Self-Report Scale. J. Atten. Disord..

[126] Kao P.-H., Ho C.-H., Huang C.L.-C. (2025). Sex differences in psychiatric comorbidities of attention-deficit/hyperactivity disorder among children, adolescents, and adults: A nationwide population-based cohort study. PLoS ONE.

[127] Frodl T., Skokauskas N. (2012). Meta-analysis of structural MRI studies in children and adults with attention deficit hyperactivity disorder indicates treatment effects. Acta Psychiatr. Scand..

[128] Valera E.M., Faraone S.V., Murray K.E., Seidman L.J. (2007). Meta-analysis of structural imaging findings in attention-deficit/hyperactivity disorder. Biol. Psychiatry.

[129] Ellison-Wright I., Ellison-Wright Z., Bullmore E. (2008). Structural brain change in Attention Deficit Hyperactivity Disorder identified by meta-analysis. BMC Psychiatry.

[130] Nakao T., Radua J., Rubia K., Mataix-Cols D. (2011). Gray matter volume abnormalities in ADHD: Voxel-based meta-analysis exploring the effects of age and stimulant medication. Am. J. Psychiatry.

[131] Qiu A., Crocetti D., Adler M., Mahone E.M., Denckla M.B., Miller M.I., Mostofsky S.H. (2009). Basal ganglia volume and shape in children with attention deficit hyperactivity disorder. Am. J. Psychiatry.

[132] Onnink A.M.H., Zwiers M.P., Hoogman M., Mostert J.C., Kan C.C., Buitelaar J., Franke B. (2014). Brain alterations in adult ADHD: Effects of gender, treatment and comorbid depression. Eur. Neuropsychopharmacol..

[133] Villemonteix T., De Brito S.A., Kavec M., Balériaux D., Metens T., Slama H., Baijot S., Mary A., Peigneux P., Massat I. (2015). Grey matter volumes in treatment naïve vs. chronically treated children with attention deficit/hyperactivity disorder: A combined approach. Eur. Neuropsychopharmacol..

[134] Dupont G., van Rooij D., Buitelaar J.K., Reif A., Grimm O. (2022). Sex-related differences in adult attention-deficit hyperactivity disorder patients—An analysis of external globus pallidus functional connectivity in resting-state functional MRI. Front. Psychiatry.

[135] Rosch K.S., Mostofsky S.H., Nebel M.B. (2018). ADHD-Related Sex Differences in Fronto-Subcortical Intrinsic Functional Connectivity and Associations with Delay Discounting. J. Neurodev. Disord..

[136] Jung B., Ahn K., Justice C., Norman L., Price J., Sudre G., Shaw P. (2022). Rare copy number variants in males and females with childhood attention-deficit/hyperactivity disorder. Mol. Psychiatry.

[137] de Jong M., Wynchank D.S.M.R., van Andel E., Beekman A.T.F., Kooij J.J.S. (2023). Female-specific pharmacotherapy in ADHD: Premenstrual adjustment of psychostimulant dosage. Front. Psychiatry.

[138] Haimov-Kochman R., Berger I. (2014). Cognitive functions of regularly cycling women may differ throughout the month, depending on sex hormone status; a possible explanation to conflicting results of studies of ADHD in females. Front. Hum. Neurosci..

[139] Roberts B., Eisenlohr-Moul T., Martel M.M. (2018). Reproductive steroids and ADHD symptoms across the menstrual cycle. Psychoneuroendocrinology.

[140] Shanmugan S., Epperson C.N. (2014). Estrogen and the prefrontal cortex: Towards a new understanding of estrogen’s effects on executive functions in the menopause transition. Hum. Brain Mapp..

[141] Young S., Adamo N., Ásgeirsdóttir B.B., Branney P., Beckett M., Colley W., Cubbin S., Deeley Q., Farrag E., Gudjonsson G. (2020). Females with ADHD: An expert consensus statement taking a lifespan approach providing guidance for the identification and treatment of attention-deficit/ hyperactivity disorder in girls and women. BMC Psychiatry.

[142] Cortese S., Adamo N., Del Giovane C., Mohr-Jensen C., Hayes A.J., Carucci S., Atkinson L.Z., Tessari L., Banaschewski T., Coghill D. (2018). Comparative efficacy and tolerability of medications for attention-deficit hyperactivity disorder in children, adolescents, and adults: A systematic review and network meta-analysis. Lancet Psychiatry.

[143] Castells X., Blanco-Silvente L., Cunill R. (2018). Amphetamines for attention deficit hyperactivity disorder (ADHD) in adults. Cochrane Database Syst. Rev..

[144] Knouse L.E., Safren S.A. (2010). Current status of cognitive behavioral therapy for adult attention-deficit hyperactivity disorder. Psychiatr. Clin. N. Am..

[145] Aoshima K.A., Banu S., Udoetuk S., Shah A.A., Moukaddam N. (2017). To Prescribe or Not? Pharmacological Options in Adult Attention-Deficit/Hyperactivity Disorder. Psychiatr. Ann..

[146] Treatment in adult ADHD|Oxford Textbook of Attention Deficit Hyperactivity Disorder|Oxford Academic. https://academic.oup.com/book/24517/chapter-abstract/187669990?redirectedFrom=fulltext.

[147] Schoeman R., de Klerk M. (2017). Adult attention-deficit hyperactivity disorder: A database analysis of South African private health insurance. S. Afr. J. Psychiatr..

[148] Thomsen P.H., Houmann T., Ishøy P.L. (2025). Pharmacological treatment of ADHD in children and adults. Ugeskr Laeger.

[149] Levin C.J., Goodman D.W., Adler L.A. (2018). Review of Cardiovascular Effects of ADHD Medications. Psychiatr. Ann..

[150] Wojnowski N.M., Zhou E., Jee Y.H. (2022). Effect of stimulants on final adult height. J. Pediatr. Endocrinol. Metab..

[151] (2024). Attention-Deficit/Hyperactivity Disorder Medications for Adults: Drugs.

[152] Santosh P.J., Sattar S., Canagaratnam M. (2011). Efficacy and tolerability of pharmacotherapies for attention-deficit hyperactivity disorder in adults. CNS Drugs.

[153] Sogard A.S., Mickleborough T.D. (2022). The therapeutic potential of exercise and caffeine on attention-deficit/hyperactivity disorder in athletes. Front. Neurosci..

[154] Kazarov C., Peasah S.K., McConnell E., Fischer K.K., Good C.B. (2024). Trends in Pediatric Attention-Deficit Hyperactive Disorder Diagnoses and Prescription Utilization: 2016 to 2019. J. Dev. Behav. Pediatr..

[155] Martinez-Raga J., Knecht C., de Alvaro R. (2015). Profile of guanfacine extended release and its potential in the treatment of attention-deficit hyperactivity disorder. Neuropsychiatr. Dis. Treat.

[156] Tan X., Xu Y., Wang S., Li J., Hu C., Chen Z., Cheng Q., Wang Z. (2023). Efficacy and Safety of SPN-812 (Extended-Release Viloxazine) in Children and Adolescents with Attention-Deficit/Hyperactivity Disorder: A Systematic Review and Meta-Analysis. Brain Sci..

[157] Kawabe K., Horiuchi F., Matsumoto Y., Inoue S., Okazawa M., Hosokawa R., Nakachi K., Soga J., Ueno S. (2024). Practical clinical guidelines and pharmacological treatment for attention-deficit hyperactivity disorder in Asia. Neuropsychopharmacol. Rep..

[158] Pan N., Ma T., Liu Y., Zhang S., Hu S., Shekara A., Cao H., Gong Q., Chen Y. (2025). Overlapping and differential neuropharmacological mechanisms of stimulants and nonstimulants for attention-deficit/hyperactivity disorder: A comparative neuroimaging analysis. Psychol. Med..

[159] Wang C.H., Mazursky-Horowitz H., Chronis-Tuscano A. (2014). Delivering Evidence-Based Treatments for Child Attention-Deficit/Hyperactivity Disorder (ADHD) in the Context of Parental ADHD. Curr. Psychiatry Rep..

[160] Stevenson C.S., Whitmont S., Bornholt L., Livesey D., Stevenson R.J. (2002). A cognitive remediation programme for adults with Attention Deficit Hyperactivity Disorder. Aust. N. Zeal. J. Psychiatry.

[161] Leaberry K.D., Rosen P.J., Fogleman N.D., Walerius D.M., Slaughter K.E. (2020). Comorbid Internalizing and Externalizing Disorders Predict Lability of Negative Emotions Among Children With ADHD. J. Atten. Disord..

[162] Prevatt F., Yelland S. (2015). An Empirical Evaluation of ADHD Coaching in College Students. J. Atten. Disord..

[163] Ing C., Mills J.P. (2017). ‘Hey, look at me’: An {auto}ethnographic account of experiencing ADHD symptoms within sport. Qual. Res. Sport, Exerc. Health.

[164] Mitchell J.T., Benson J.W., Knouse L.E., Kimbrel N.A., Anastopoulos A.D. (2013). Are Negative Automatic Thoughts Associated with ADHD in Adulthood?. Cogn. Ther. Res..

[165] Waxmonsky J.G., Waschbusch D.A., Belin P., Li T., Babocsai L., Humphery H., Pariseau M.E., Babinski D.E., Hoffman M.T., Haak J.L. (2016). A Randomized Clinical Trial of an Integrative Group Therapy for Children With Severe Mood Dysregulation. J. Am. Acad. Child Adolesc. Psychiatry.

[166] López-Pinar C., Martínez-Sanchís S., Carbonell-Vayá E., Sánchez-Meca J., Fenollar-Cortés J. (2020). Efficacy of Nonpharmacological Treatments on Comorbid Internalizing Symptoms of Adults With Attention-Deficit/Hyperactivity Disorder: A Meta-Analytic Review. J. Atten. Disord..

[167] Gaur S., Pallanti S. (2020). Treatment Outcomes in an Adult Attention Deficit Hyperactivity Disorder Clinic With a Focus on Executive Functioning and Sluggish Cognitive Tempo. Cureus.

[168] Seery C., Cochrane R.H., Mulcahy M., Kilbride K., Wrigley M., Bramham J. (2025). “A one-stop shop”: Real-world use and app-users’ experiences of a psychoeducational smartphone app for adults with ADHD. Internet Interv..

[169] Weusten L.H., Heijnen-Kohl S.M.J., Ellison J., van Alphen S.P.J. (2014). Interference of attention-deficit hyperactivity disorder in an older adult with a severe personality disorder and dermatillomania. Int. Psychogeriatrics.

[170] Kiraz S., Sertçelik S. (2021). Adult attention deficit hyperactivity disorder and early maladaptive schemas. Clin. Psychol. Psychother..

[171] Wietecha L.A., Clemow D.B., Buchanan A.S., Young J.L., Sarkis E.H., Findling R.L. (2016). Atomoxetine Increased Effect over Time in Adults with Attention-Deficit/Hyperactivity Disorder Treated for up to 6 Months: Pooled Analysis of Two Double-Blind, Placebo-Controlled, Randomized Trials. CNS Neurosci. Ther..

[172] Knouse L.E., Teller J., Brooks M.A. (2017). Meta-analysis of cognitive–behavioral treatments for adult ADHD. J. Consult. Clin. Psychol..

[173] Strålin E.E., Thorell L.B., Lundgren T., Bölte S., Bohman B. (2025). Cognitive behavioral therapy for ADHD predominantly inattentive presentation: Randomized controlled trial of two psychological treatments. Front. Psychiatry.

[174] U.S. Food & Drug Administration Drugs@FDA: FDA-Approved Drugs—New Drug Application 021977; VYVANSE (Lisdexamfetamine Dimesylate). https://www.accessdata.fda.gov/scripts/cder/daf/index.cfm?event=overview.process&ApplNo=021977.

[175] U.S. Food & Drug Administration Janssen-Cilag Manufacturing, LLC. CONCERTA® (Methylphenidate HCl) Extended-Release Tablets—Prescribing Information, Label Edition 2015; Application No. 021121, Supplement s035. https://www.accessdata.fda.gov/drugsatfda_docs/label/2015/021121s035lbl.pdf.

[176] U.S. Food & Drug Administration (2002). Eli Lilly and Company. STRATTERA® (Atomoxetine Hydrochloride) Capsules—Prescribing Information, NDA 21-411 Package Insert. https://www.accessdata.fda.gov/drugsatfda_docs/label/2002/21411_strattera_lbl.pdf.

[177] U.S. Food & Drug Administration (2021). Adhansia XR® (methylphenidate hydrochloride) Extended-Release Capsules—Prescribing Information, NDA 211964 s000. https://www.accessdata.fda.gov/drugsatfda_docs/label/2021/211964s000lbl.pdf.

[178] Drug Safety Communications (2025). FDA. https://www.fda.gov/drugs/drug-safety-and-availability/drug-safety-communications.

[179] Pinto S., Correia-De-Sá T., Sampaio-Maia B., Vasconcelos C., Moreira P., Ferreira-Gomes J. (2022). Eating Patterns and Dietary Interventions in ADHD: A Narrative Review. Nutrients.

[180] Catalá-López F., Hutton B., Núñez-Beltrán A., Page M.J., Ridao M., Saint-Gerons D.M., A Catalá M., Tabarés-Seisdedos R., Moher D. (2017). The pharmacological and non-pharmacological treatment of attention deficit hyperactivity disorder in children and adolescents: A systematic review with network meta-analyses of randomised trials. PLoS ONE.

[181] De Crescenzo F., Cortese S., Adamo N., Janiri L. (2017). Pharmacological and non-pharmacological treatment of adults with ADHD: A meta-review. Évid. Based Ment. Health.

[182] Pelsser L., Frankena K., Toorman J., Pereira R.R. (2020). Retrospective Outcome Monitoring of ADHD and Nutrition (ROMAN): The Effectiveness of the Few-Foods Diet in General Practice. Front. Psychiatry.

[183] Khoshbakht Y., Moghtaderi F., Bidaki R., Hosseinzadeh M., Salehi-Abargouei A. (2021). The effect of dietary approaches to stop hypertension (DASH) diet on attention-deficit hyperactivity disorder (ADHD) symptoms: A randomized controlled clinical trial. Eur. J. Nutr..

[184] Renoux C., Shin J., Dell’ANiello S., Fergusson E., Suissa S. (2016). Prescribing trends of attention-deficit hyperactivity disorder (ADHD) medications in UK primary care, 1995–2015. Br. J. Clin. Pharmacol..

[185] Li L., Zhu N., Zhang L., Kuja-Halkola R., D’onofrio B.M., Brikell I., Lichtenstein P., Cortese S., Larsson H., Chang Z. (2024). ADHD Pharmacotherapy and Mortality in Individuals With ADHD. JAMA.

[186] Kenter R.M.F., Gjestad R., Lundervold A.J., Nordgreen T. (2023). A self-guided internet-delivered intervention for adults with ADHD: Results from a randomized controlled trial. Internet Interv..

[187] Seery C., Leonard-Curtin A., Naismith L., King N., O’DOnnell F., Byrne B., Boyd C., Kilbride K., Wrigley M., McHugh L. (2024). Feasibility of the Understanding and Managing Adult ADHD Programme: Open-access online group psychoeducation and acceptance and commitment therapy for adults with attention-deficit hyperactivity disorder. BJPsych Open.

[188] Bozzatello P., Rocca P., Mantelli E., Bellino S. (2019). Polyunsaturated Fatty Acids: What is Their Role in Treatment of Psychiatric Disorders?. Int. J. Mol. Sci..

[189] Landaas E.T., Aarsland T.I.M., Ulvik A., Halmøy A., Ueland P.M., Haavik J. (2016). Vitamin levels in adults with ADHD. BJPsych Open.

[190] A Gordon H., Rucklidge J.J., Blampied N.M., Johnstone J.M. (2015). Clinically Significant Symptom Reduction in Children with Attention-Deficit/Hyperactivity Disorder Treated with Micronutrients: An Open-Label Reversal Design Study. J. Child Adolesc. Psychopharmacol..

[191] French B., Nalbant G., Wright H., Sayal K., Daley D., Groom M.J., Cassidy S., Hall C.L. (2024). The impacts associated with having ADHD: An umbrella review. Front. Psychiatry.

[192] Cherkasova M.V., Roy A., Molina B.S., Scott G., Weiss G., Barkley R.A., Biederman J., Uchida M., Hinshaw S.P., Owens E.B. (2022). Review: Adult Outcome as Seen Through Controlled Prospective Follow-up Studies of Children With Attention-Deficit/Hyperactivity Disorder Followed Into Adulthood. J. Am. Acad. Child Adolesc. Psychiatry.

[193] Biederman J., Faraone S.V. (2006). The Effects of Attention-Deficit/Hyperactivity Disorder on Employment and Household Income. MedGenMed.

[194] Adamou M., Arif M., Asherson P., Aw T.-C., Bolea B., Coghill D., Guðjónsson G., Halmøy A., Hodgkins P., Müller U. (2013). Occupational issues of adults with ADHD. BMC Psychiatry.

[195] Ahlberg R., Du Rietz E., Ahnemark E., Andersson L.M., Werner-Kiechle T., Lichtenstein P., Larsson H. (2023). Real-life instability in ADHD from young to middle adulthood: A nationwide register-based study of social and occupational problems. BMC Psychiatry.

[196] Young S., Morris R., Toone B., Tyson C. (2007). Planning ability in adults with attention-deficit/hyperactivity disorder. Neuropsychology.

[197] Young S., Morris R., Toone B., Tyson C. (2006). Spatial working memory and strategy formation in adults diagnosed with attention deficit hyperactivity disorder. Pers. Individ. Differ..

[198] Fuermaier A.B.M., Tucha L., Butzbach M., Weisbrod M., Aschenbrenner S., Tucha O. (2021). ADHD at the workplace: ADHD symptoms, diagnostic status, and work-related functioning. J. Neural Transm..

[199] Oscarsson M., Nelson M., Rozental A., Ginsberg Y., Carlbring P., Jönsson F. (2022). Stress and work-related mental illness among working adults with ADHD: A qualitative study. BMC Psychiatry.

[200] Kubik J.A. (2010). Efficacy of ADHD coaching for adults with ADHD. J. Atten. Disord..

[201] Kahveci Öncü B., Tutarel Kişlak Ş. (2022). Marital Adjustment and Marital Conflict in Individuals Diagnosed with ADHD and Their Spouses. Noro Psikiyatr Ars.

[202] Ginapp C.M., Greenberg N.R., Macdonald-Gagnon G., Angarita G.A., Bold K.W., Potenza M.N. (2023). The experiences of adults with ADHD in interpersonal relationships and online communities: A qualitative study. SSM-Qual. Res. Health.

[203] Mokrova I., O’Brien M., Calkins S., Keane S. (2010). Parental ADHD Symptomology and Ineffective Parenting: The Connecting Link of Home Chaos. Parenting.

[204] Støre S.J., Van Zalk N., Schwartz W.G., Nilsson V., Tillfors M. (2024). The Relationship Between Social Anxiety Disorder and ADHD in Adolescents and Adults: A Systematic Review. J. Atten. Disord..

[205] Nyström A., Petersson K., Janlöv A.-C. (2020). Being Different but Striving to Seem Normal: The Lived Experiences of People Aged 50+ with ADHD. Issues Ment. Health Nurs..

[206] Young S., Klassen L.J., Reitmeier S.D., Matheson J.D., Gudjonsson G.H. (2023). Let’s Talk about Sex… and ADHD: Findings from an Anonymous Online Survey. Int. J. Environ. Res. Public Health.

[207] Cortese S., Kelly C., Chabernaud C., Proal E., Di Martino A., Milham M.P., Castellanos F.X. (2012). Toward systems neuroscience of ADHD: A meta-analysis of 55 fMRI studies. Am. J. Psychiatry.

[208] Hoogman M., Bralten J., Hibar D.P., Mennes M., Zwiers M.P., Schweren L.S.J., van Hulzen K.J.E., Medland S.E., Shumskaya E., Jahanshad N. (2017). Subcortical brain volume differences in participants with attention deficit hyperactivity disorder in children and adults: A cross-sectional mega-analysis. Lancet Psychiatry.

[209] Oades R.D., Dauvermann M.R., Schimmelmann B.G., Schwarz M.J., Myint A.-M. (2010). Attention-deficit hyperactivity disorder (ADHD) and glial integrity: S100B, cytokines and kynurenine metabolism-effects of medication. Behav. Brain Funct..

[210] Thapar A., Cooper M., Eyre O., Langley K. (2013). Practitioner Review: What have we learnt about the causes of ADHD?. J. Child Psychol. Psychiatry.

